# The Relationship Between Ferritin and BMI is Mediated by Inflammation Among Women in Higher-Income Countries, But Not in Most Lower-Income Countries Nor Among Young Children: A Multi-Country Analysis

**DOI:** 10.1093/cdn/nzac139

**Published:** 2022-09-09

**Authors:** Jennie N Davis, Anne Williams, Charles D Arnold, Fabian Rohner, James P Wirth, Yaw Addo, Rafael C Flores-Ayala, Brietta M Oaks, Melissa F Young, Parminder S Suchdev, Reina Engle-Stone

**Affiliations:** University of California, Davis Department of Nutrition, Institute for Global Nutrition, Davis, CA, USA; University of Otago, Department of Human Nutrition, Dunedin, New Zealand; University of California, Davis Department of Nutrition, Institute for Global Nutrition, Davis, CA, USA; GroundWork, Fläsch, Switzerland; GroundWork, Fläsch, Switzerland; Division of Nutrition, Physical Activity and Obesity, Centers for Disease Control and Prevention, Chamblee, GA, USA; Rollins School of Public Health, Emory University, Atlanta, GA, USA; Division of Nutrition, Physical Activity and Obesity, Centers for Disease Control and Prevention, Chamblee, GA, USA; Department of Nutrition and Food Sciences, University of Rhode Island, Kingston, RI, USA; Global Health Department, Emory University, Atlanta, GA, USA; Department of Pediatrics and Global Health, Emory University, Atlanta, GA, USA; Division of Global Health Protection, Centers for Disease Control and Prevention, Atlanta, GA, USA; University of California, Davis Department of Nutrition, Institute for Global Nutrition, Davis, CA, USA

**Keywords:** ferritin, inflammation, acute phase proteins, iron status, assessment, biomarker, overweight, obesity

## Abstract

**Background:**

In the presence of inflammation, the serum or plasma ferritin concentration (“ferritin” hereafter) transiently increases, confounding its interpretation as an iron status marker. The extent to which adiposity-related inflammation may influence ferritin interpretation is uncertain.

**Objectives:**

We describe relationships between weight status, inflammation, and ferritin among nonpregnant women of reproductive age (WRA; 15–49 years) and preschool-age children (PSC; 6–59 months) with normal weight to overweight or obesity (OWOB) in differing geographic settings.

**Methods:**

Cross-sectional data were separately analyzed from 18 surveys (WRA) and 25 surveys (PSC) from the Biomarkers Reflecting Inflammation and Nutritional Determinants of Anemia (BRINDA) project, excluding observations with underweight, wasting, pregnancy, or malaria. Relationships were assessed between BMI (in WRA) or BMI-for-age z-score (BAZ; in PSC), inflammatory biomarkers of C-reactive protein (CRP) and/or α-1-acid glycoprotein (AGP), ferritin by linear regression, and potential mediation by CRP and/or AGP in relationships between BMI or BAZ and ferritin with structural equation modeling. Regression and mediation models accounted for complex survey designs. Results were grouped by World Bank income classifications.

**Results:**

In 5 of 6 surveys among WRA from upper-middle and high-income countries, ferritin was significantly positively associated with BMI, and this relationship was partially (or fully, in the United States) mediated by CRP and/or AGP. Mediation was present in 4 of 12 surveys for WRA in low- and lower-middle income countries. Among PSC, ferritin was positively associated with CRP and/or AGP in all surveys, but there were no significant CRP- or AGP-mediated relationships between ferritin and BAZ, except a negative relationship in the Philippines.

**Conclusions:**

Where having OWOB is common among WRA, measurements of inflammatory biomarkers and their uses in interpreting ferritin may improve iron status assessments. While these relationships were inconsistent among PSC, inflammation was common and should be measured to interpret iron status. Included Kenyan trial data are registered at clinicaltrials.gov as NCT01088958.

## Introduction

Epidemiological studies of health and nutrition status frequently measure plasma or serum ferritin concentrations (hereafter referred to as “ferritin”) to evaluate a population's iron status (1). However, the presence of inflammation confounds iron status assessments by transiently increasing ferritin concentrations, potentially resulting in erroneous underestimates of iron deficiency (2). Thus, to accurately assess and interpret the ferritin results of a population assessment, researchers should measure inflammation biomarkers, identify potential sources of inflammation, and consider their influences on iron status assessments.

Sources of inflammation often differ by setting. For example, inflammation due to malarial illness or diarrheal disease may be more common in low-income countries (LIC) and lower-middle-income countries (LMIC) (3). In contrast, inflammation associated with having overweight and obesity (OWOB) may be more common in upper-middle income countries (UMIC) and high-income countries (HIC) (4), though OWOB are increasingly prevalent across global contexts (5, 6). Inflammation is the body's physiological response to injury, illness, infection, or environmental insult, and is characterized by the presence of proinflammatory cytokines and acute-phase proteins (APP), such as C-reactive protein (CRP) and α-1-acid glycoprotein (AGP) (4). In obesity, excess adipose tissue—in particular, visceral adipose tissue—releases proinflammatory cytokines and CRP, which then stimulate the production and release of APPs from hepatocytes, macrophages, and others, creating an environment of prolonged, low-grade, systemic inflammation (7, 8). Persistent inflammation may increase the risk of iron deficiency due to sustained disruptions to intestinal iron absorption and systemic iron distribution (9).

Research examining the relationship between iron status and inflammation due to illness and infection has largely concentrated on data from LIC and LMIC. This is, in part, because of the direct interaction of iron and pathogens (e.g., malaria, helminths) associated with common illnesses in many of these countries (10–13). In contrast, literature examining adiposity and inflammation and adiposity and iron deficiency has largely been carried out in populations from UMIC and HIC (14–18). Much of the research on adiposity and iron status has yielded mixed results in the magnitude or direction of association of this relationship due to variations in evaluation methods (19, 20), populations assessed (21, 22), and the degree of iron deficiency at the time of assessment (18, 23). As the obesity prevalence increases globally, research exploring the combined influence of inflammation and adiposity on iron status is needed in countries with and without a high infectious disease burden.

The Biomarkers Reflecting Inflammation and Nutritional Determinants of Anemia (BRINDA) project previously presented analyses of national survey data from multiple countries highlighting the influence of inflammation in iron status assessments in different populations (24). The BRINDA project also established a statistical method to adjust for inflammation when measuring iron status (2, 25–27), an approach adopted by the WHO in its most recent guidelines on the use of ferritin to assess iron statuses of populations (1). Additionally, the BRINDA project recently reported the prevalences and independence of intraindividual OWOB and iron deficiency among adult women and young children (28, 29) and determined that in both groups, OWOB was not associated with iron deficiency based on inflammation-adjusted ferritin. However, this analysis estimated iron deficiency with inflammation-adjusted ferritin, and did not examine whether the relationships between BMI and iron status indicators may be influenced by inflammation.

Therefore, we analyzed data from the BRINDA project to explore the following objectives: *1*) to determine the relationships between weight status (BMI), inflammation (CRP and/or AGP), and ferritin among adult women and young children with normal weight to OWOB in differing geographic settings; and *2*) to examine whether inflammation mediates the relationships between BMI (in women) or BMI-for-age z-score (BAZ; in children) and ferritin in these same settings.

## Methods

### Data source and inclusion criteria

Using secondary, deidentified data from nationally or regionally representative surveys from the BRINDA project, we analyzed 18 data sets from 17 countries with data on nonpregnant women of reproductive age (WRA; 15–49 years) and 25 data sets from 22 countries with data on preschool-aged children (PSC; 6–59 month). The criteria for survey inclusion in the BRINDA project, data set harmonization, and methodology of anthropometric calculations and biochemical collection have been previously documented (28–31). For all surveys, all participants provided informed consent, referrals were made for severe anemia and/or severe acute malnutrition, participants did not directly benefit from their participation in the survey, and participants were not informed of the results of the current study. The Kenyan data included in BRINDA were part of a clinical trial registered at clinicaltrials.gov as NCT01088958.

Our inclusion criteria matched those of 2 previous analyses examining the intraindividual double burden of malnutrition among PSC (28) and WRA (29). Additional inclusion criteria specific to the present analyses were surveys that included a marker of inflammation (CRP and/or AGP) and measured serum or plasma ferritin. As the present analyses examined relationships between BMI, ferritin, and inflammation among populations with normal weight to OWOB, observations with BMI values < 18.5 kg/m^2^ (in WRA), or BAZ or weight-for-height z-score (WHZ) values < −2 SD (in PSC) were excluded due to concerns that individuals with underweight or wasting were likely to have inflammation from other sources, such as infectious diseases. Other excluded observations were those with 0 values for ferritin; those from WRA who were pregnant or whose height or weight was outside the ranges of 101.6–219.9 cm and 22.7–222.2 kg, respectively (29); those from PSC with BAZ values less than −5 SD or greater than 5 SD (28); and those with a positive malaria result [to minimize the influence of inflammation from infectious disease; *n* = 4 (in WRA) and *n* = 8 (in PSC) surveys measuring malaria]. Observations with other morbidity symptoms (i.e., reported fever or diarrhea) were not excluded because the variables were not reported uniformly across surveys, and prior BRINDA analyses showed that reported morbidity was not consistently associated with inflammation (32). We did not apply a sample-size cutoff for excluding surveys after the application of the exclusion criteria; however, we did ensure the analytical sample sizes met the criteria for a mediation analysis (33). BAZ and WHZ values were recalculated for the BRINDA database using the WHO growth standards (30). The proportions of observations excluded overall and by individual exclusion criterion are presented in **Supplemental Tables 1** and **2**.

### Variable definitions

The outcome variables were unadjusted ferritin (µg/L), CRP (mg/L), and AGP (g/L); the predictor variables were BMI (kg/m^2^; in WRA) or BAZ (in PSC), CRP (mg/L), and AGP (g/L). For consistency with prior BRINDA analyses (28), we applied BAZs to all age groups, though WHZ values are recommended for use for children < 24 months (34). Ferritin was measured in all surveys. CRP was measured in all WRA surveys and 21 PSC surveys, AGP was measured in 11 WRA surveys and 18 PSC surveys, and both CRP and AGP were measured in 11 WRA surveys and 15 PSC surveys. For the mediation analyses, the outcome variable was ferritin, the predictor variable was BMI or BAZ, and the mediating variables were CRP and/or AGP.

Covariates were defined for each survey as reported by the survey representative, unless otherwise indicated (30): age (years in WRA or months in PSC); sex (in PSC only); urban residence (compared to rural residence); high household socioeconomic status (SES; the ordinal 3-category SES variable from the harmonized BRINDA data set (30) was dichotomized into a binary variable of low SES versus high SES, where high SES included both medium and high categories); access to an improved water source (compared to no access or access only to an unimproved water source); access to an improved toilet (compared to no access or access only to an unimproved toilet); and high education level (compared to no education or primary school only), measured as the respondent education level in WRA or maternal education level in PSC, except in surveys that reported household-head education level (Burkina Faso for PSC and WRA; Colombia for PSC; Mexico in 2006 for PSC and WRA; and the United States for PSC). Covariates with more than 2 categories (SES, water, sanitation, and education level) were dichotomized for ease of interpretation.

The following variables described the nutrition and health status of the survey populations: BMI (in WRA), categorized as normal (18.5–24.9 kg/m^2^), overweight (25.0–29.9 kg/m^2^), or obese (≥30.0 kg/m^2^); BAZ (in PSC), categorized as normal (−2 to 2 SD), overweight (>2 SD to ≤3 SD), and obese (>3 SD); any inflammation, defined as CRP > 5 mg/L and/or AGP > 1 g/L; and iron deficiency, defined as inflammation-adjusted ferritin <15 µg/L (in WRA) or <12 µg/L (in PSC).

### Statistical analyses

Data were analyzed using SAS version 9.4 (SAS Institute) and STATA version 16 (StataCorp). All analyses accounted for complex survey designs (cluster and strata) by calculating variance estimates and applying survey weights, except Mongolia, which used simple random sampling. Analyses were conducted separately by survey and by population using continuous variables available in each data set. A *P* value < 0.05 was considered to be statistically significant. Covariates available for analyses are listed by survey in **Supplemental Table 3**.

Means (95% CIs) are presented for continuous variables, except CRP, AGP, and ferritin are presented as geometric means (95% CIs) due to their nonnormal distributions and age is presented as the median (IQR), Percentages (95% CIs) are presented for categorical variables. For descriptive analyses, ferritin and iron deficiency were adjusted for inflammation following the BRINDA regression correction approach (2, 30). Previous BRINDA work has examined the impact of this adjustment on estimates of iron deficiency prevalences, and found the prevalences increased after adjustment (2, 25, 26).

### Bivariate and multivariable analyses

In surveys for which >30% of the analytical sample values had a single low value (representing the lower limit of detection in the lab analyses), we generated and applied a random number between 0 and the lowest value (Afghanistan for PSC, Colombia for WRA and PSC, Georgia for PSC, and Zambia for PSC). For example, more than half of the CRP values in the surveys from Colombia were reported as 0.2 [WRA *n* = 4838 (58% of the analytical sample); PSC: *n* = 2476 (66% of the analytical sample)]; for these observations, we generated and applied a random value between 0 and 0.2 for CRP (35, 36) and confirmed that the directions and strengths of associations did not differ with randomly generated values by rerunning all regression models with multiple random seeds. For all surveys, ferritin, CRP, and AGP were natural-log (*ln*) transformed to achieve normal distributions and the residuals were visually examined. For any surveys where the distribution of residuals appeared abnormal after transformation of the outcome variables, a sensitivity analysis compared the Spearman's rank correlation coefficient with the bivariate linear regression estimate, and the results were examined to determine the appropriateness of continuing with linear regression analyses. All regression analyses were conducted with the unadjusted ferritin variable.

We assessed the following bivariate linear regression models in each survey:

1. Y(*ln*Ferritin) = B_0_ + B_1_(BMI)
2. Y(*ln*CRP) = B_0_ + B_1_(BMI)
3. Y(*ln*AGP) = B_0_ + B_1_(BMI)
4. Y(*ln*Ferritin) = B_0_ + B_1_(*ln*CRP)
5. Y(*ln*Ferritin) = B_0_ + B_1_(*ln*AGP)

For models 1–3, the PSC calculations used BAZ in place of BMI.

Relationships between the previously defined covariates and outcome variables were assessed in separate bivariate linear regression analyses, with marginally significant covariates (*P* < 0.10) included in multivariable-adjusted models. Models were assessed for collinearity with variance inflation factors (>5) and tolerance (>0.1). CRP and AGP variables were analyzed in separate models, rather than combined into a single variable, in order to examine the individual associations between CRP or AGP with iron and inflammation status. As prespecified in our analysis plan, all bivariate WRA analyses were also completed in stratified analyses by age (15–29 years and 30–49 years), as inflammation due to chronic disease has been associated with age (37). For consistency with prior BRINDA analyses, PSC surveys were stratified separately by age (6–23 months and 24–59 months) and by sex (28).

Results are presented grouped by World Bank country-income-level classifications (LIC, LMIC, UMIC, HIC) according to their ranking at the time the survey was conducted (38). While the country groupings do not imply that the results may be generalized by income classification, the groupings have historically represented differences in potential sources of inflammation among populations with regard to noncommunicable and communicable disease burdens (39, 40). We also examined heterogeneity among surveys to decide whether to proceed with pooling by income classification. Within each income level, we tested for heterogeneity by constructing pooled regression models using the individual-level data from each survey and evaluating the interactions between the survey and predictor variables.

### Mediation analyses

**FIGURE 1:**
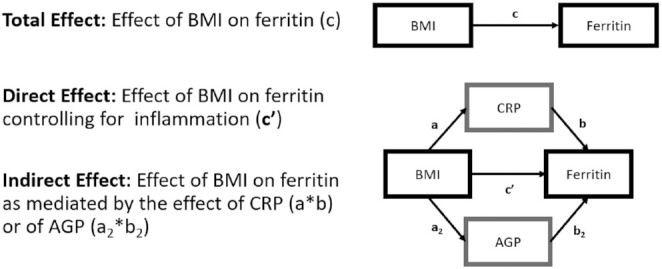
Mediation analysis path diagram. Ferritin, CRP, and AGP were analyzed as their *natural-log* equivalents and as continuous variables. Ferritin refers to either the serum or plasma ferritin concentration (41). AGP, α-1-acid glycoprotein; CRP, C-reactive protein.

We used mediation analyses to test the hypothesis that inflammation mediates part or all of the relationship between BMI or BAZ and ferritin among WRA or PSC with a normal to elevated BMI or BAZ, respectively. Mediation analyses were conducted using structural equation modeling procedures in STATA according to the path diagram, with the path through CRP or AGP eliminated in surveys without the variable (**Figure 1**). Mediation was considered present if both the total effect (the effect of BMI or BAZ on ferritin) and the indirect effect (the effect of BMI or BAZ on ferritin as mediated by the effect of CRP and/or AGP) were significant at a *P* value < 0.05 (41, 42). Linearized SEs and 95% CIs were generated and adjusted for the complex survey design by calculating variance estimates and applying survey weights. Final mediation estimates were exponentiated, and adjusted mediation results are presented as percentage changes in ferritin concentrations for every 1-unit change in BMI or BAZ. Survey-specific structural-equation-modeling models were adjusted for the same marginally significant covariates found in bivariate models. All mediation analyses met the minimum sample size requirement of at least 10 observations per linear relation (i.e., *n* = 40 observations for models with CRP or AGP only; *n* = 50 observations for models with both CRP and AGP) (33).

### Sensitivity analyses

We conducted sensitivity analyses by including BMI values < 18.5 kg/m^2^ (in WRA) or BAZ or WHZ values < −2 SD (in PSC) for surveys where greater than 50% of the observations were excluded due to our prespecified exclusion criteria (*n* = 5 surveys for WRA; *n* = 8 surveys for PSC). Sensitivity mediation analyses were also conducted in data sets with available malaria data (*n* = 4 surveys for WRA; *n* = 8 surveys for PSC) to examine the inflammation-mediated relationships between BMI or BAZ and ferritin among all individuals versus among those without a positive malaria result. An additional sensitivity mediation analysis was conducted in a subset of surveys to assess the effects of excluding observations with reported morbidity symptoms [fever or diarrhea in the past 24 hours (Kenya in 2010 for PSC) or past 2 weeks (Liberia for PSC; Côte d'Ivoire and Malawi for WRA)]; results were compared qualitatively.

### Ethical approval and human subject research protocol

The BRINDA protocol was reviewed by the institutional review board of the NIH and was deemed to be non–human subjects research.

## Results

### Participant characteristics

Among WRA, the analytical sample size ranged from 61 (Burkina Faso) and 147 (India) to 8300 (Colombia), with a total sample size of 33,429. The median age was approximately 30 years. The proportion of the analytical sample residing in rural areas ranged from 20.8% (Mexico in 2012) to 89.7% (Malawi); the Indian and Nigerian surveys were conducted in rural areas only. In surveys with SES data (*n* = 14), more than 60% of the population was classified as having a high SES, except in Mexico (in 2006), where the high-SES proportion was 53% (**Supplemental Table 4**).

Among PSC, the analytical sample size ranged from 63 (Burkina Faso) to 5824 (Pakistan), with a total sample size of 28,727. Most surveys (*n* = 13) included children 6–59 months of age, while other surveys included the following age ranges: Bangladesh (in 2010) included children aged 6–24 months; Kenya (in 2007 and 2010), Liberia, and Mongolia included children aged 6–35 months; Vietnam included children aged 10–59 months; Cameroon, Georgia, and Mexico (in 2006 and 2012) included children aged 12–59 months; and Burkina Faso and the Philippines included children aged 24–59 months. In the analytical sample, more than 80% of participants resided in rural areas in Cambodia, Laos, Malawi, and the Philippines; the surveys from Kenya (in 2007 and 2010) and Nigeria were conducted in rural areas only. Among surveys that measured SES (*n* = 18), the proportion of the analytical sample classified as having a low SES ranged from 5.1% (Afghanistan) to 84.0% (Philippines; **Supplemental Table 5**).

### Prevalences of OWOB, iron deficiency, and inflammation among WRA

Overall, the prevalence of OWOB among WRA was generally greater in UMIC and HIC than in LMIC and LIC, with the greatest prevalence in Mexico in 2012 (UMIC; 72%; 95% CI: 69.0, 75.1). The prevalences of inflammation-adjusted iron deficiency among WRA ranged from 20.8% to 44.3% in UMIC and HIC and from 2.0% to 63.9% in LIC and LMIC. The prevalences of inflammation (elevated CRP and/or AGP) ranged from 16.0% to 34.9% among surveys from UMIC and HIC and from 7.1% to 74.5% among surveys from LIC and LMIC (**Table 1**).

**TABLE 1:** Biological and nutritional characteristics for women of reproductive age (15–49 years) with normal weight to overweight or obesity by survey in the BRINDA project^1^

CIC^2^	Country, survey year	*n*	BMI, kg/m2, mean (95% CI)	Overweight or obesity,^3^ % (95% CI)	CRP, mg/L mean (95% CI)	AGP, g/L mean (95% CI)	Any inflammation,^4^ % (95% CI)	Ferritin,^5^ µg/L mean (95% CI)	Iron deficiency,^6^ % (95% CI)
Low	Afghanistan, 2013	571	25.1 (24.6, 25.6)	42.0 (35.9, 48.1)	0.7 (0.5, 0.8)	0.7 (0.7, 0.7)	18.1 (13.9, 22.4)	24.1 (20.4, 27.8)	30.2 (23.3, 37.1)
	Burkina Faso, 2010	61	20.8 (20.0, 21.7)	3.1 (0.0, 7.8)	1.8 (1.3, 2.2)	1.2 (1.1, 1.3)	74.5 (63.5, 85.5)	31.9 (25.6, 38.3)	14.0 (2.9, 25.1)
	Cambodia, 2014	609	22.9 (22.5, 23.3)	22.5 (17.1, 27.9)	0.8 (0.7, 0.9)	0.7 (0.7, 0.8)	35.9 (25.9, 45.8)	57.8 (54.5, 61.1)	3.5 (1.8, 5.2)
	Côte d'Ivoire, 2007	706	23.5 (23.1, 23.8)	26.0 (22.3, 29.8)	1.5 (1.3, 1.7)	0.8 (0.8, 0.8)	31.9 (27.9, 35.9)	26.7 (24.4, 29.0)	23.4 (19.6, 27.3)
	Laos, 2006	690	22.4 (22.0, 22.8)	17.1 (12.5, 21.7)	0.4 (0.3, 0.5)	0.7 (0.7, 0.7)	14.1 (10.8, 17.4)	28.9 (23.3, 34.5)	28.7 (22.0, 35.5)
	Malawi, 2016	594	22.5 (22.1, 23.0)	17.2 (13.3, 22.0)	0.7 (0.6, 0.8)	0.6 (0.6, 0.6)	11.7 (8.4, 15.1)	33.0 (30.3, 35.7)	14.1 (10.3, 17.9)
Low-middle	Cameroon, 2009	594	24.7 (24.3, 25.1)	39.0 (34.4, 43.6)	0.9 (0.8, 1.1)	0.7 (0.7, 0.8)	16.3 (13.2, 19.4)	25.7 (24.3, 27.0)	19.2 (15.6, 22.7)
	Georgia, 2009	1605	26.0 (25.6, 26.3)	46.3 (43.2, 49.5)	2.2 (2.0, 2.4)	—	30.6 (27.5, 33.7)	99.2 (93.9,104.4)	2.0 (1.1, 3.0)
	India, 2011	147	21.5 (20.9, 22.0)	12.9 (6.2, 19.7)	0.5 (0.3, 0.6)	0.8 (0.8, 0.8)	21.1 (15.4, 26.8)	10.8 (9.3, 12.3)	63.9 (57.8, 70.1)
	Nigeria, 2012	506	24.2 (23.6, 24.7)	35.2 (29.5, 40.9)	1.6 (1.4, 1.8)	0.7 (0.7, 0.8)	25.7 (21.6, 29.8)	31.3 (28.3, 34.3)	17.0 (12.8, 21.2)
	Pakistan, 2011	5004	24.4 (24.2, 24.5)	36.5 (34.6, 38.3)	1.0 (0.9, 1.0)	0.8 (0.8, 0.8)	32.5 (30.8, 34.3)	18.4 (17.8, 19.0)	42.1 (40.3, 44.0)
	Vietnam, 2010	1178	21.7 (21.6, 21.9)	10.0 (8.2, 11.8)	0.9 (0.8, 0.9)	—	7.1 (5.9, 8.4)	37.6 (34.6, 40.7)	17.9 (15.1, 20.7)
Upper-middle	Azerbaijan, 2013	2528	26.8 (26.5, 27.1)	57.3 (54.7, 59.8)	1.1 (1.1, 1.2)	0.9 (0.9, 0.9)	34.9 (32.5, 37.4)	16.2 (15.3, 17.1)	44.3 (41.7, 46.8)
	Colombia, 2010	8300	25.2 (25.1, 25.3)	44.4 (43.0, 45.8)	0.4 (0.4, 0.4)	—	22.1 (20.9, 23.4)	26.2 (25.5, 26.9)	24.4 (23.1, 25.6)
	Mexico, 2006	2910	27.7 (27.4, 28.1)	65.3 (62.4, 68.3)	1.9 (1.8, 2.0)	—	24.8 (22.2, 27.3)	18.0 (16.7, 19.3)	35.8 (32.7, 38.9)
	Mexico, 2012	3540	28.7 (28.3, 29.1)	72.0 (69.0, 75.1)	1.8 (1.7, 2.0)	—	21.2 (18.6, 23.9)	15.3 (14.3, 16.3)	42.7 (39.3, 46.1)
High	United Kingdom, 2014	862	26.5 (25.9, 27.1)	49.6 (44.5, 54.8)	2.2 (2.1, 2.4)	—	16.0 (12.8, 19.2)	23.5 (21.6, 25.4)	28.9 (24.4, 33.3)
	United States, 2006	3024	27.9 (27.4, 28.5)	56.3 (52.7, 59.9)	1.8 (1.7, 1.9)	—	26.3 (24.1, 28.5)	28.6 (27.5, 29.8)	20.8 (18.7, 22.8)

1 CRP, AGP, and ferritin values are presented as geometric means (95% CIs) due to nonnormal distributions. All estimates account for survey design variables (cluster, strata, weight). A dash (—) indicates the variable was unavailable in that survey. The inclusion criteria were having a BMI ≥ 18.5 kg/m^2^, not being pregnant, and having a negative malaria test result. AGP, α-1-acid glycoprotein; BRINDA, Biomarkers Reflecting Inflammation and Nutritional Determinants of Anemia; CIC, country income classification; CRP, C-reactive protein.

2 CIC was defined according to the World Bank definition for the year in which the survey was conducted (38).

3 Having overweight or obesity was defined as having a BMI ≥ 25.0 kg/m^2^.

4 Any inflammation was defined as CRP > 5 mg/L or AGP > 1 g/L.

5 Ferritin was measured in plasma or serum, as reported in the survey.

6 Iron deficiency was defined as having a serum or plasma ferritin concentration < 15 µg/L, adjusted for inflammation using the BRINDA regression correction approach (2).

### Prevalences of OWOB, iron deficiency, and inflammation among PSC

Among PSC, the prevalence of OWOB was ≤11.6% across all surveys from LIC and LMIC, except in Georgia (19.6%; 95% CI: 16.7%, 22.6%). Among UMIC and HIC, Azerbaijan had the greatest prevalence of OWOB, at 15.6% (95% CI: 12.7%, 18.5%). Across income classifications and world regions, the prevalences of inflammation-adjusted iron deficiency among PSC varied, ranging from 0.6% (95% CI: 0.2%, 1.0%) in Georgia to >60% in Kenya (in 2007 and 2010), Nicaragua, and Pakistan. The prevalences of any inflammation ranged from 10.7% to 92.4% among surveys from LIC and LMIC and from 6.1% to 30.8% among UMIC and HIC (**Table 2**).

**TABLE 2:** Biological and nutritional characteristics for preschool-aged children (6–59 months) with normal weight to overweight or obesity by survey in the BRINDA project^1^

CIC^2^	Country, survey year	*n*	BAZ, mean (95% CI)	Overweight or obesity,^3^ % (95% CI)	CRP, mg/L mean (95% CI)	AGP, g/L mean (95% CI)	Any inflammation,^4^ % (95% CI)	Ferritin,^5^ µg/L, mean (95% CI)	Iron deficiency,^6^ % (95% CI)
Low	Afghanistan, 2013	595	0.2 (0.1, 0.3)	7.1 (3.8, 10.3)	0.3 (0.2, 0.3)	0.8 (0.8, 0.8)	10.7 (7.1, 14.3)	21.7 (18.9, 24.4)	30.3 (25.8, 34.9)
	Bangladesh, 2010	1179	−0.6 (−0.7, −0.5)	2.1 (1.3, 2.9)	0.8 (0.6, 0.9)	0.9 (0.9, 0.9)	34.4 (30.8, 38.1)	23.8 (22.2, 25.4)	25.1 (21.7, 28.6)
	Bangladesh, 2012	368	−0.4 (−0.6, −0.2)	4.3 (1.1, 7.6)	0.7 (0.6, 0.9)	0.8 (0.8, 0.9)	27.8 (20.0, 35.5)	25.0 (21.7, 28.3)	18.8 (11.2, 26.4)
	Burkina Faso, 2010	63	−0.1 (−0.5, 0.3)	1.5 (0, 5.2)	4.9 (2.9, 6.9)	1.6 (1.5, 1.7)	92.4 (87.0, 97.7)	32.2 (25.1, 39.3)	16.3 (0.9, 31.8)
	Cambodia, 2014	599	−0.4 (−0.5, −0.4)	0.8 (0, 1.7)	0.6 (0.5, 0.7)	0.8 (0.7, 0.9)	38.5 (30.5, 46.5)	43.8 (41.5, 46.1)	8.0 (5.8, 10.1)
	Côte d'Ivoire, 2007	435	0.1 (0.0, 0.2)	6.2 (3.7, 8.6)	2.0 (1.6, 2.4)	1.1 (1.1, 1.1)	59.3 (54.3, 64.3)	15.4 (14.0, 16.8)	48.6 (44.0, 53.3)
	Kenya, 2007	665	0.2 (0.2, 0.3)	4.2 (2.5, 5.9)	1.0 (0.8, 1.2)	1.1 (1.1, 1.1)	59.4 (54.6, 64.2)	7.0 (6.5, 7.6)	80.0 (76.7, 83.3)
	Kenya, 2010	551	0.3 (0.2, 0.3)	4.5 (3.0, 6.1)	0.9 (0.7, 1.1)	1.0 (0.9, 1.0)	47.9 (42.0, 53.8)	10.7 (9.8, 11.7)	64.4 (59.9, 69.0)
	Laos, 2006	443	−0.2 (−0.3, −0.1)	0.7 (0, 1.7)	0.5 (0.4, 0.7)	0.9 (0.9, 1.0)	43.2 (35.7, 50.8)	21.2 (18.7, 23.8)	32.1 (27.5, 36.6)
	Liberia, 2011	956	−0.1 (−0.2, 0)	2.5 (1.4, 3.7)	1.4 (1.2, 1.6)	1.0 (0.9, 1.0)	50.4 (45.9, 54.8)	12.3 (11.4, 13.2)	58.4 (54.1, 62.7)
	Malawi, 2016	748	0.1 (−0.1, 0.2)	5.3 (2.9, 7.8)	1.0 (0.8, 1.2)	1.0 (1.0, 1.1)	49.0 (42.5, 55.6)	24.2 (21.2, 27.2)	25.7 (18.4, 33.1)
	Mongolia, 2006	239	0.8 (0.7, 0.9)	10.5 (6.9, 15.1)	—	0.8 (0.8, 0.8)	24.7 (19.4, 30.7)	11.9 (10.4, 13.4)	55.7 (49.1, 62.1)
	Nicaragua, 2005	946	−0.9 (−1.0, −0.7)	5.1 (3.5, 6.8)	—	0.8 (0.8, 0.9)	26.9 (21.3, 32.6)	13.4 (12.3, 14.5)	60.1 (54.3, 65.9)
	Zambia, 2009	330	0.4 (0.3, 0.6)	6.1 (2.8, 9.3)	1.5 (0.8, 2.1)	1.0 (0.9, 1.0)	70.3 (66.5, 74.1)	27.8 (25.0, 30.6)	24.2 (16.0, 32.5)
Low-middle	Cameroon, 2009	556	0.4 (0.3, 0.5)	4.2 (2.0, 6.3)	1.3 (1.1, 1.5)	0.9 (0.9, 0.9)	37.7 (32.7, 42.7)	17.3 (16.0, 18.5)	37.6 (33.5, 41.7)
	Georgia, 2009	2064	1.1 (1.0, 1.1)	19.6 (16.7, 22.6)	0.9 (0.8, 1.1)	—	24.6 (21.7, 27.5)	124.3 (118.9,129.8)	0.6 (0.2, 1.0)
	Nigeria, 2012	303	0.4 (0.2, 0.7)	11.6 (5.9, 17.2)	2.0 (1.5, 2.4)	1.0 (0.9, 1.0)	56.1 (49.1, 63.1)	22.6 (20.5, 24.7)	25.4 (19.0, 31.9)
	Pakistan, 2011	5824	−0.1 (−0.1, 0)	5.5 (4.8, 6.2)	—	0.9 (0.9, 0.9)	35.6 (34.0, 37.2)	11.7 (11.3, 12.2)	59.7 (58.1, 61.4)
	Philippines, 2011	1656	−0.1 (−0.2, 0)	2.1 (1.0, 3.3)	0.7 (0.6, 0.9)	0.8 (0.8, 0.8)	25.9 (22.3, 29.4)	14.8 (13.8, 15.7)	45.0 (41.4, 48.6)
	Vietnam, 2010	344	−0.1 (−0.3, 0)	4.1 (2.0, 6.2)	0.7 (0.6, 0.8)	—	11.9 (9.2, 14.6)	24.9 (22.6, 27.1)	24.4 (19.5, 29.3)
Upper-middle	Azerbaijan, 2013	987	0.9 (0.8, 1.0)	15.6 (12.7, 18.5)	0.3 (0.3, 0.4)	0.8 (0.8, 0.9)	30.8 (27.0, 34.6)	20.4 (19.1, 21.7)	29.1 (25.0, 33.2)
	Colombia, 2010	7753	0.4 (0.4, 0.4)	3.9 (3.1, 4.7)	0.6 (0.6, 0.7)	—	18.8 (17.1, 20.5)	23.5 (22.7, 24.2)	21.5 (19.8, 23.3)
	Mexico, 2006	1562	0.6 (0.5, 0.6)	7.6 (5.9, 9.2)	0.6 (0.6, 0.7)	—	11.1 (8.9, 13.4)	14.3 (13.3, 15.3)	45.8 (42.0, 49.6)
	Mexico, 2012	2454	0.6 (0.5, 0.6)	8.4 (6.6, 10.2)	0.5 (0.4, 0.6)	—	11.8 (9.4, 14.3)	18.2 (17.4, 18.9)	30.3 (27.3, 33.2)
High	United States, 2006	1081	0.6 (0.5, 0.7)	9.1 (6.8, 11.5)	0.3 (0.3, 0.4)	—	6.1 (4.5, 7.8)	21.4 (20.4, 22.4)	22.9 (19.2, 26.6)

1 CRP, AGP, and ferritin values are presented as geometric means (95% CIs) due to nonnormal distributions. All estimates account for survey design variables (cluster, strata, weight), except Mongolia, which followed a simple random sampling design. A dash (—) indicates the variable was unavailable in that survey. The inclusion criteria were having a BAZ or WHZ ≥ −2 SD and a negative malaria test result. AGP, α-1-acid glycoprotein; BAZ, BMI-for-age z-score; BRINDA, Biomarkers Reflecting Inflammation and Nutritional Determinants of Anemia; CIC, Country Income Classification; CRP, C-reactive protein; WHZ, weight-for-height z-score.

2 CIC was defined according to the World Bank definition for the year in which the survey was conducted (38).

3 Having overweight or obesity was defined as having a BAZ of ≥ 2 SD.

4 Any inflammation was defined as a CRP > 5 mg/L or AGP > 1 g/L.

5 Ferritin was measured in plasma or serum, as reported in the survey.

6 Iron deficiency was defined as having a serum or plasma ferritin concentration < 12 µg/L, adjusted for inflammation using the BRINDA regression correction approach (2).

### Associations between BMI, ferritin, and inflammation among WRA

Results from adjusted linear models that examined the relationships between BMI and ferritin varied among income classifications (**Supplemental Table 6**). Having a greater BMI was consistently and significantly associated with having a greater ferritin concentration in all surveys from UMIC and HIC (*n* = 6: Azerbaijan, Colombia, Mexico in 2006 and in 2012, the United Kingdom, and the United States), and in 4 of 12 surveys from LIC and LMIC (Cambodia, Laos, Georgia, and Pakistan). Among UMIC and HIC, the percentage increases in ferritin concentration for every 1-unit increase in BMI ranged from 1.0% (95% CI: 0.3%, 1.7%; United States) to 3.5% (95% CI: 2.5%, 4.4%; Azerbaijan); among the 4 LIC and LMIC surveys, they ranged from 0.8% (95% CI: 0.1%, 1.5%; Pakistan) to 5.5% (95% CI: 2.4%, 8.7%; Laos). BMI was also significantly positively associated with inflammation (CRP and/or AGP) across all surveys, except those in Afghanistan, Burkina Faso, and Nigeria (Supplemental Table 6). Ferritin was significantly positively associated with inflammation across all surveys, except in Malawi, where the relationship was positive but nonsignificant (CRP only; Supplemental Table 6). Stratified results by age are presented in **Supplemental Table 7**; however, no obvious trends emerged.

### Associations between BAZ, ferritin, and inflammation among PSC

Among PSC, the relationships between BAZ and ferritin varied across surveys and income classifications in the adjusted models (**Supplemental Table 8**). BAZ was significantly negatively associated with ferritin in 5 of 19 surveys from LIC and LMIC (Bangladesh in 2010, Nicaragua, Pakistan, the Philippines, and Vietnam) and 2 of 5 surveys from UMIC and HIC (Mexico in 2012 and the United States). Among these surveys, the percentage changes in ferritin concentrations for every 1-unit increase in BMI ranged from −3.5% (95% CI: −6.2%, −0.8; Pakistan) to −11.8% (95% CI: −17.9, −5.3%; Vietnam). BAZ was significantly positively associated with ferritin in Colombia. In the majority of other surveys (*n* = 11), the relationship between BAZ and ferritin was negative but nonsignificant. BAZ was significantly positively associated with CRP in the surveys from Malawi (LIC) and the United States (HIC). In all other surveys (*n* = 23), the relationship between BAZ and inflammation (CRP and/or AGP) was nonsignificant and the direction of association varied. Across all surveys, greater ferritin was significantly associated with greater inflammation, except in Georgia. In Afghanistan, only the relationship with CRP was significant, and in Burkina Faso only the relationship with AGP was significant (Supplemental Table 8). Stratified results by age and sex are presented in **Supplemental Tables 9** and **10**. There were variations by age in relationships between inflammation and BAZ and between ferritin and inflammation in some surveys. In the surveys from Cambodia, Côte d'Ivoire, Nicaragua, and Nigeria, the relationships between inflammation and BAZ changed from negative to positive as age increased.

### Mediation analyses between ferritin, BMI, and inflammation

**FIGURE 2:**
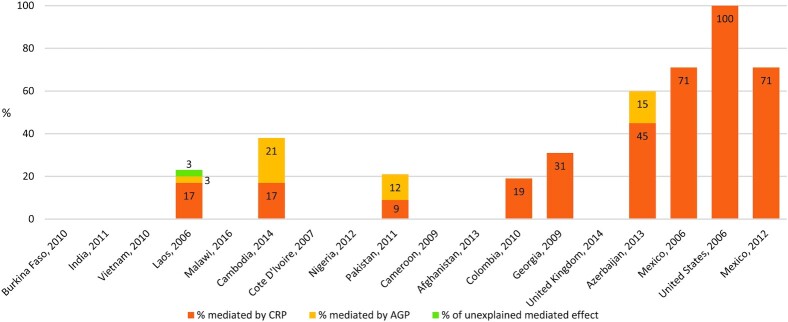
Percentages of adjusted relationships between ferritin concentrations and BMI mediated by CRP and/or AGP among WRA (15–49 years) with normal weight to overweight or obesity in 18 surveys in order of ascending mean BMI (left to right) from the BRINDA Project. Surveys with mediated effects are displayed with the percentage indicating the proportion mediated by CRP or AGP or unexplained (3% in Laos). Surveys without data indicate no mediation was observed. Both CRP and AGP were measured in 4 of the 10 surveys with mediation: Laos, Cambodia, Pakistan, and Azerbaijan. Ferritin concentrations were measured in serum or plasma, as reported in the survey. Covariates available for adjustment were age, education level (respondent or household head), household socioeconomic status, access to an improved water source, access to an improved toilet, and urban or rural residence. Covariates were included in mediation models if they were associated with the outcome variable at *P* < 0.1 in bivariate models (Supplemental Table 6). The inclusion criteria were having a BMI ≥ 18.5 kg/m^2^, not being pregnant, and having a negative malaria test result. AGP, α-1-acid glycoprotein; BRINDA, Biomarkers Reflecting Nutritional Determinants of Anemia; CRP, C-reactive protein; WRA, women of reproductive age.

In 9 of 18 surveys included in the WRA analyses, inflammation partially and positively mediated the relationships between ferritin and BMI in adjusted models (**Figure 2**; **Table 3**). Among LIC and LMIC surveys with mediation by inflammation, the average percentage of the total effect mediated by inflammation was 27%. Among UMIC and HIC, the surveys in Azerbaijan and Mexico (in 2006 and 2012) had similar mediated effects of 60% to 70%, while Colombia's mediated effect was 19%. In the United States, 100% of the total effect was mediated by inflammation. Among 4 surveys which measured both CRP and AGP, CRP accounted for >50% of the mediated effects in Laos and Azerbaijan, but AGP accounted for >50% of the mediated effects in Cambodia and Pakistan. No mediation was present in the survey from Vietnam after adjusting for age.

**TABLE 3:** Relationships between ferritin and BMI, as mediated by inflammation, among WRA (15–49 years) with normal weight to overweight or obesity by survey in the BRINDA project^1^

CIC^2^	Country, survey year	*n*	WRA mediation analyses, adjusted^3^					
			Total effect	Direct effect	Indirect effect	Mediated, %	Mediated by CRP, %	Mediated by AGP, %
Low	Afghanistan, 2013	571	−2.1 (−4.3, 0.1)	−2.7 (−4.7, −0.8)	0.6 (−0.02, 1.3)	NM	—	—
	Burkina Faso, 2010	61	−2.9 (−15.9, 10.1)	5.1 (−6.1, 17.2)	−7.5 (−12.5, −3.2)	NM	—	—
	Cambodia, 2014	609	3.8 (2.0, 5.5)	2.3 (0.4, 4.1)	1.4 (0.2, 2.6)	38%	17%	21%
	Côte d'Ivoire, 2007	706	0.5 (−1.1, 2.1)	−0.8 (−2.3, 0.7)	1.3 (0.7, 1.8)	NM	—	—
	Laos, 2006	690	11.9 (8.3, 14.3)	9.1 (5.5, 11.9)	2.6 (1.2, 3.9)	23%^4^	17%	3%
	Malawi, 2016	594	−0.5 (−2.5, 1.6)	−1.1 (−3.2, 0.1)	0.6 (0.04, 1.2)	NM	—	—
Low-middle	Cameroon, 2009	594	0.1 (−1.2, 1.4)	−0.6 (−1.9, 0.7)	0.7 (0.4, 1.0)	NM	—	—
	Georgia, 2009	1605	1.2 (0.6, 1.8)	0.8 (0.2, 1.4)	0.4 (0.1, 0.6)	31%	31%	—
	India, 2011	147	7.5 (0.07, 15.4)	3.7 (−2.1, 9.4)	3.6 (1.1, 6.2)	NM^5^	—	—
	Nigeria, 2012	506	−1.5 (−3.2, 0.3)	−1.5 (−3.2, 0.2)	0.003 (−0.04, 0.5)	NM	—	—
	Pakistan, 2011	5004	1.0 (0.4, 1.7)	0.8 (0.2, 1.5)	0.2 (0.1, 0.3)	21%	9%	11%
	Vietnam, 2010	1178	2.4 (−0.2, 4.9)	−0.5 (−3.2, 2.2)	2.9 (2.0, 3.8)	NM	—	—
Upper-middle	Azerbaijan, 2013	2528	3.5 (2.7, 4.3)	1.4 (0.1, 2.3)	2.1 (1.6, 2.6)	60%	45%	15%
	Colombia, 2010	8300	2.0 (1.4, 2.6)	1.6 (1.0, 2.2)	0.4 (0.2, 0.5)	19%	19%	—
	Mexico, 2006	2910	1.2 (0.1, 2.3)	0.4 (−0.7, 1.5)	0.9 (0.3, 1.5)	71%	71%	—
	Mexico, 2012	3540	2.7 (1.3, 4.1)	0.8 (−1.0, 2.4)	1.9 (1.2, 2.6)	71%	71%	—
High	United Kingdom, 2014	862	1.9 (0.5, 3.3)	1.3 (−1.0, 2.9)	0.6 (−0.04, 1.2)	NM	—	—
	United States, 2006	3024	1.3 (0.6, 2.0)	−0.03 (−1.0, 0.08)	1.3 (1.0, 1.7)	100%	100%	—

1 Ferritin, CRP, and AGP variables were natural-log transformed for analysis due to nonnormal distributions. Mediation estimates were exponentiated, and results are presented as the percentage changes (95% CIs) in ferritin for every 1-unit change in BMI. Ferritin concentrations were measured in serum or plasma, as reported in the survey. All estimates account for the cluster survey design (cluster, strata) with survey weights applied. The inclusion criteria were having a BMI ≥ 18.5 kg/m^2^, not being pregnant, and having a negative malaria test result. AGP, alpha-1-acid glycoprotein; BRINDA, Biomarkers Reflecting Inflammation and Nutritional Determinants of Anemia; CIC, country income classification; CRP, C-reactive protein; NM, no mediation; WRA, women of reproductive age.

2 CIC was defined according to the World Bank definition for the year in which the survey was conducted (38).

3 Model for mediation analyses:

$ln{\rm{Ferritin}} = {{\rm{\beta }}_0} + {{\rm{\beta }}_1}( {{\rm{BMI}}} ) + {M_1}( {ln{\rm{CRP}}} )[ { + {M_2}( {ln{\rm{AGP}}} )} ]$ (1)

Here, all values were continuous and AGP was included as a mediator only in analyses for which it was available in the data set. Interpretation was as follows: total effect is the effect of BMI on ferritin; direct effect is the effect of BMI on ferritin, controlling for inflammation; and indirect effect is the effect of BMI on ferritin, as mediated by the effect of CRP or AGP. Mediation was considered present when both the total and indirect effects were significant (41). The covariates available for adjustment were age, education level (respondent or household head), household socioeconomic status, access to an improved water source, access to an improved toilet, and urban or rural residence. Covariates were included in the mediation model if they were associated with the outcome variable at a *P* value < 0.1 in bivariate models (Supplemental Table 6). Unadjusted mediation estimates are presented in Supplemental Table 11.

4 In the survey from Laos, 23% of the relationship between BMI and ferritin was mediated by inflammation, with 17% of the mediated effect through CRP, 3% of the mediated effect through AGP, and 3% of the mediated effect unexplained.

5 For the survey from India, the CI for the total effect appears significant; however, as the *P* value was 0.063, mediation was not considered to be present.

No significant mediated relationships emerged among PSC, except in the Philippines, where the mediated relationship was negative, suggesting inconsistent mediation (−2%; 95% CI: −3.7, −0.2; **Table 4**). Unadjusted mediation results for both WRA and PSC are presented in **Supplemental Table 11**.

**TABLE 4:** Relationships between ferritin BAZs as mediated by inflammation among PSC (6–59 months) with normal weight to overweight or obesity by survey in the BRINDA project^1^

CIC^2^	Country, survey year	*n*	PSC mediation analyses, adjusted^3^					
			Total effect	Direct effect	Indirect effect	Mediated, %	Mediated by CRP, %	Mediated by AGP, %
Low	Afghanistan, 2013	595	−2.8 (−11.7, 6.0)	−1.9 (−10.8, 7.0)	−0.9 (−2.8, 1.1)	NM	—	—
	Bangladesh, 2010	1179	−6.5 (−11.0, −2.3)	−5.7 (−10.1, −1.6)	−0.8 (−2.2, 0.5)	NM	—	—
	Bangladesh, 2012	368	−4.8 (−15.3, 5.5)	−2.7 (−12.6, 7.1)	−2.2 (−6.1, 1.7)	NM	—	—
	Burkina Faso, 2010	63	−10.5 (−41.9, 19.8)	−10.6 (−37.8, 15.4)	0.1 (−7.5, 7.7)	NM	—	—
	Cambodia, 2014	599	−6.2 (−16.6, 3.8)	−7.0 (−16.6, 2.0)	0.9 (−3.3, 5.0)	NM	—	—
	Côte d'Ivoire, 2007	435	1.4 (−7.2, 10.0)	−0.6 (−8.6, 7.4)	2.0 (−2.2, 6.2)	NM	—	—
	Kenya, 2007	665	−7.3 (−−16.8, 1.6)	−7.2 (−16.0, 1.1)	−0.1 (−2.7, 2.5)	NM	—	—
	Kenya, 2010	551	−5.0 (−13.4, 3.2)	−7.3 (−15.0, −0.2)	2.5 (−1.8, 6.7)	NM	—	—
	Laos, 2006	443	−5.8 (−18.2, 6.2)	−4.5 (−17.2, 7.9)	−1.3 (−5.0, 2.3)	NM	—	—
	Liberia, 2011	956	−1.6 (−6.9, 3.7)	−1.7 (−7.2, 3.8)	0.1 (−1.9, 2.1)	NM	—	—
	Malawi, 2016	748	−3.0 (−16.4, 10.4)	−1.5 (−8.5, 5.4)	1.3 (−1.5, 4.0)	NM	—	—
	Mongolia, 2006	239	−0.2 (−7.2, 6.7)	−1.9 (−15.0, 11.2)	−1.1 (−3.9, 1.7)	NM	—	—
	Nicaragua, 2005	946	−9.0 (−17.2, −1.7)	−9.5 (−16.8, −3.1)	0.5 (−1.4, 2.4)	NM	—	—
	Zambia, 2009	330	−8.6 (−20.1, 2.0)	−9.2 (−20.0, 0.8)	0.6 (−4.1, 5.4)	NM	—	—
Low-middle	Cameroon, 2009	556	12.9 (4.6, 19.7)	12.5 (4.9, 18.7)	0.3 (−1.8, 2.5)	NM	—	—
	Georgia, 2009	2064	0.3 (−2.5, 3.1)	0.3 (−2.5, 3.1)	−0.02 (−0.1, 0.1)	NM	—	—
	Nigeria, 2012	303	−2.6 (−10.8, 5.5)	−3.8 (−13.2, 5.5)	1.2 (−2.2, 4.7)	NM	—	—
	Pakistan, 2011	5824	−3.0 (−5.6, −0.5)	−3.1 (−5.8, −0.6)	0.1 (−0.1, 0.3)	NM	—	—
	Philippines, 2011	1656	−6.2 (−12.6, −0.1)	−4.3 (−10.7, 1.9)	−2.0 (−3.7, −0.2)	31%	8%	23%
	Vietnam, 2010	344	−11.8 (−19.4, −5.8)	−11.1 (−19.1, −4.3)	−0.9 (−2.5, 0.7)	NM	—	—
Upper-middle	Azerbaijan, 2013	987	−2.1 (−6.9, 2.7)	−0.1(−4.6, 4.3)	−2.0 (−4.2, 0.2)	NM	—	—
	Colombia, 2010	7753	−4.8 (−7.9, −1.9)	−4.7 (−7.8, −1.8)	−0.2 (−0.6, 0.3)	NM	—	—
	Mexico, 2006	1562	−3.2 (−9.0, 2.5)	−3.4 (−9.3, 2.4)	0.2 (−1.3, 1.8)	NM	—	—
	Mexico, 2012	2454	−5.8 (−9.6, −2.3)	−6.5 (−10.3, −3.2)	0.8 (−0.3, 1.8)	NM	—	—
High	United States, 2006	1081	−5.8 (−9.6, −2.3)	−6.5 (−10.3, −3.2)	0.8 (−0.3, 1.8)	NM	—	—

1 Ferritin, CRP, and AGP variables were natural-log transformed for analysis due to nonnormal distributions. Mediation estimates were exponentiated, and results are presented as percentage changes (95% CIs) in ferritin for every 1-unit change in BAZ. Ferritin concentrations were measured in serum or plasma, as reported in the survey. All estimates account for a cluster survey design (cluster, strata) with survey weights applied, except in the survey from Mongolia, which used simple random sampling. The inclusion criteria were having a BAZ or WHZ ≥ −2 SD and a negative malaria test result. AGP, alpha-1-acid glycoprotein; BAZ, BMI-for-age z-score; BRINDA, Biomarkers Reflecting Inflammation and Nutritional Determinants of Anemia; CIC, country income classification; CRP, C-reactive protein; NM, no mediation; PSC, pre-school age children; WHZ, weight-for-height z-score.

2 CIC was defined according to the World Bank definition for the year in which the survey was conducted (38).

3 Model for mediation analyses:

$ln{\rm{Ferritin}} = {{\rm{\beta }}_0} + {{\rm{\beta }}_1}( {{\rm{BAZ}}} ) + {M_1}( {ln{\rm{CRP}}} )[ { + {M_2}( {ln{\rm{AGP}}} )} ]$ (2)

Here, all values were continuous and AGP was included as a mediator only in analyses for which it was available in the data set. Interpretation was as follows: total effect is the effect of BAZ on ferritin; direct effect is the effect of BAZ on ferritin, controlling for inflammation; and indirect effect is the effect of BAZ on ferritin as mediated by the effect of CRP or AGP. Mediation was considered present when both the total and indirect effects were significant (41). The covariates available for adjustment were age, education level (respondent or maternal education level), household socioeconomic status, access to an improved water source, access to an improved toilet, and urban or rural residence. Covariates were included in the mediation model if they were associated with the outcome variable at a *P* value < 0.1 in bivariate models (Supplemental Table 6). Unadjusted mediation estimates are presented in Supplemental Table 11.

### Sensitivity analyses

Overall, pooled analyses and generations of pooled estimates were not possible due to extensive heterogeneity in all WRA and PSC surveys, with the exception of 1 model (CRP = BMI) for the WRA LIC pooled grouping (pooled estimate β = −0.02; *P* = 0.2; data not presented). Among surveys that measured the malaria status (*n* = 4 for WRA; *n* = 8 for PSC), results from the sensitivity mediation analyses that included all observations, regardless of malaria test result, were similar to the main mediation results (**Supplemental Table 12**). Results were also similar to the main results in sensitivity analyses that included all BMI values < 18.5 kg/m^2^ (in WRA) or BAZ or WHZ values < −2 SD (in PSC) in surveys where greater than 50% of the observations were excluded due toour prespecified exclusion criteria, and among surveys with the additional excluded morbidity data of reported fever or diarrhea (data not presented).

## Discussion

We explored relationships between BMI or BAZ, inflammation, and ferritin concentrations in 18 data sets from 17 countries with data on WRA and 25 data sets from 22 countries with data on PSC, with data drawn from both high- and low-income contexts, according to World Bank classifications. We found that for WRA with BMIs > 18.5 kg/m^2^ residing in UMIC and HIC, greater ferritin was associated with having a greater BMI, and this relationship could be partially (or fully in the case of the US survey) explained by the inflammatory markers of CRP or AGP. This pattern was present in fewer surveys for WRA in LIC and LMIC, and was often not mediated by the presence of inflammation. Among PSC, greater ferritin was significantly associated with greater inflammation in all but 2 surveys; however, significant associations between ferritin and BAZ and inflammation and BAZ were not observed. Additionally, neither CRP nor AGP mediated the relationships between ferritin and BAZ among children, except in the Philippines, where the relationship was negative.

Our findings suggest that in settings where OWOB are common among WRA, measurements of inflammatory biomarkers and their use in interpreting ferritin concentrations may improve iron status assessments, even where infections are less common or less severe. These findings may also be helpful in clinical settings, as the ferritin concentration is the most commonly used measurement of iron status (43–46). While some clinical guidelines suggest including a measure of inflammation to interpret the iron status when inflammatory conditions are present, OWOB is often not highlighted as contributing to inflammation (43–46). Further research is needed for individual patient care in clinical settings, as cases of iron deficiency may be missed among WRA with OWOB if inflammation is not measured or accounted for in interpretations of ferritin concentrations. Additional consideration of the impacts of inflammation on iron status assessments and potential iron deficiency may be necessary in individuals with anemia of chronic disease (37, 47).

Among WRA, the prevalence of OWOB was greater than 20% in the majority of surveys where inflammation mediated the relationship between ferritin and BMI, suggesting that inflammation associated with adiposity may influence iron status assessments. This is consistent with literature suggesting that adiposity and inflammation—primarily measured by CRP—are strongly correlated in WRA (48–50), which may explain our result from the US survey, where inflammation (measured only with CRP in this survey) explained 100% of the inflammatory effect. In our analyses, where mediation was present and both CRP and AGP were measured (WRA only: Azerbaijan, Cambodia, Laos, and Pakistan), AGP explained 3% to 21% of the inflammatory effect in each case. Previous literature suggests that CRP and AGP reflect different phases of the inflammatory acute-phase response, with CRP levels rising and falling quickly after the initial insult and AGP levels rising later and staying elevated longer, potentially indicating longer-term or chronic inflammation (51). This framework does not translate easily to OWOB or other chronic conditions where inflammation is not the response to a single infection, and is not consistent with observations of associations between OWOB and CRP. Moreover, a study comparing the kinetics of CRP and AGP found similar inflammatory response patterns between the 2 proteins in relation to ferritin (52). Further research that includes AGP and other APPs and adipocytokines may aid in understanding which are most useful for iron assessments, as inflammatory biomarkers, such as IL-6 and α-1-antitrypsin, have been shown to be important in characterizing inflammation associated with obesity (4, 7, 49). Measurements of hepcidin may also provide insight into the corresponding effects on the iron metabolism.

Among PSC, little evidence of mediation was present, and we did not see much of a relationship between BAZ and inflammation, likely due to the overall low prevalence of OWOB across PSC surveys. Although our results indicate that having OWOB was not strongly associated with inflammation among PSC, we observed a strong relationship between inflammation and ferritin, which likely indicates a burden of inflammation from causes other than OWOB, and underscores the need to measure inflammation when assessing iron status among PSC. An additional consideration is that many countries globally are not on track to meet the World Health Assembly nutrition target for 2025 to prevent increases in child OWOB (5, 53), which compels increased monitoring of indicators of body composition and inflammation in this group. Literature examining iron deficiency, inflammation, and adiposity among WRA and PSC suggests adiposity could be a risk factor for iron deficiency and, thus, iron deficiency should be monitored, particularly in countries with rapidly increasing prevalences of OWOB (14, 54).

We grouped our findings by country income classifications to aid interpretation of findings by likely sources of inflammation (i.e., greater BMI and lower prevalence of infections in UMIC or HIC), under the assumption that inflammation due to common infectious illnesses, such as malaria or helminths, would be more common in LIC and LMIC, while inflammation due to OWOB would be more common in UMIC and HIC (4). We recognize that sources of inflammation and patterns of malnutrition are not uniform across or within these groupings. However, the groupings did allow for a pattern to emerge among WRA: inflammation mediated the relationship between BMI and ferritin in all 4 UMIC surveys (Azerbaijan, Colombia, and Mexico in 2006 and 2012). One interpretation of these findings is that these countries have high prevalences of OWOB alongside persistent but decreasing prevalences of infectious diseases (e.g., malaria and diarrhea) that lead to inflammation (39). Similarly, CRP entirely explained the relationship between ferritin and BMI among WRA in the United States, where infectious illnesses prevalent in LIC and LMIC are less common but the OWOB prevalence is high. Thus, the interpretation of iron status in the context of inflammation is necessary in settings of high BMI, such as HIC, as is continued surveillance of iron deficiency, as chronic inflammation may increase the risk of iron deficiency over time (37). This recommendation is in line with literature examining the likelihood of overlapping iron deficiency and OWOB across global settings (55–59). We did not find that inflammation mediated the relationship between ferritin and BMI in the UK survey, the only other survey in an HIC, though the survey prevalence of OWOB was 50% and that of iron deficiency was 29%. We found no obvious explanation for this observation, which warrants further investigation.

A limitation of our analyses was the use of BMI alone as an indicator of adiposity, though other direct measures, such as waist circumference, waist-to-hip ratio, or skinfold thickness have been found to be highly correlated with BMI when predicting cardiometabolic risks (60). Of greater consideration is the relation of inflammation to the distribution of adipose tissue, as abdominal obesity has been found to be strongly related to inflammation among adult women (61, 62) and among children and adolescents (16, 63), and has a significant influence on iron status (62). Additionally, we were limited in our choice of inflammatory markers to CRP and AGP. For example, in the Colombia survey, the low levels of inflammation made it difficult to assess the relationships between CRP and other factors. Though CRP and AGP are often measured in health surveys (64, 65), different biomarkers, such as the APP IL-6 or the hepatic hormone hepcidin, have also been reported as being elevated among individuals with obesity (57, 62), and their inclusion may help further characterize the relationships between iron status markers and inflammation.

Another limitation is the loss of sample sizes in some surveys due to our exclusion criteria. Though our exclusion criteria were selected *a priori* to align with our research objective, in some surveys, such as India (WRA only), Burkina Faso (WRA and PSC), and Afghanistan (WRA and PSC), the exclusion of participants with underweight or wasting and those that tested positive for malaria resulted in >80% losses of sample sizes. The criteria also excluded all participants residing in urban settings in the surveys from India (WRA), Nigeria (WRA and PSC), and Kenya (PSC in both survey years). For these surveys, we interpreted the results with caution. Additionally, the reduced sample may have biased some results towards the null hypothesis if a large proportion of the analytical sample was excluded. To examine the potential effects the loss of sample sizes had on our outcomes, we conducted sensitivity mediation analyses that included all malaria observations, with results similar to our main results. We also conducted sensitivity analyses that included all observations categorized as having underweight or being wasted in surveys where >50% of the observations were excluded; these observations were originally excluded to avoid U-shaped distributions (and violation of regression assumptions), as conditions of underweight are also inflammatory. Sensitivity analyses that included these observations did not change the analysis linearity and, in retrospect, their exclusion may not have been necessary. However, their exclusion allowed us to focus our analyses on our populations of interest: those with normal weight to OWOB. Finally, the cross-sectional nature of the data prevented temporal interpretations.

In conclusion, it has become standard to include measures of inflammation to interpret iron statuses in large nutrition and health surveys in LIC and LMIC, mainly due to the role of infections in systemic inflammation, but the roles of adiposity and its associated inflammation in iron status assessments has been less clear. Our findings suggest that adiposity may affect iron status assessments, particularly among WRA in countries where the prevalence of OWOB is high, an observation that has implications for both public health and clinical settings. These results suggest that inflammation should be measured and considered alongside ferritin in assessments of iron statuses in contexts where OWOB is common, even if inflammation related to infections is expected to be low. While the results were not consistent with adiposity-related inflammation influencing iron status assessments among children, inflammation was nevertheless common among children and should continue to be measured to interpret iron statuses. With the nutrition transition persisting in LIC and LMIC and the prevalence of OWOB increasing across the globe (5, 40), the contribution of adiposity-related inflammation may become more important, and other potential sources of inflammation beyond infections likewise merit exploration.

## Supplementary Material

nzac139_Supplemental_File

## Data Availability

Data described in the manuscript, code book, and analytic code will be made available upon request pending approval from the BRINDA steering committee and country representatives.

## Notes

This study was supported by the Bill & Melinda Gates Foundation (Grant No. INV-010744), Centers for Disease Control and Prevention, Eunice Kennedy Shriver National Institute of Child Health and Human Development, HarvestPlus, and the United States Agency for International Development.

Author disclosures: The authors report no conflicts of interest.

Supplemental Tables 1–12 are available from the “Supplementary data” link in the online posting of the article and from the same link in the online table of contents at https://academic.oup.com/cdn/.

Abbreviations: AGP, α-1-acid glycoprotein; APP, acute-phase protein; BAZ, BMI-for-age z-score; BRINDA, Biomarkers Reflecting Inflammation and Nutritional Determinants of Anemia; CRP, C-reactive protein; HIC, high-income country; LIC, low-income country; LMIC, lower-middle-income country; OWOB, overweight or obesity; PSC, preschool-age children; SES, socioeconomic status; UMIC, upper-middle-income country; WHZ, weight-for-height z-score; WRA, women of reproductive age.
