# Supplementary material for: The Relationship Between Ferritin and BMI is Mediated by Inflammation Among Women in Higher-Income Countries, But Not in Most Lower-Income Countries Nor Among Young Children: A Multi-Country Analysis
Source: Curr Dev Nutr. 2022 Sep 9;6(10):nzac139. doi: 10.1093/cdn/nzac139 (PMC9718651; doi:10.1093/cdn/nzac139)
Supplement: nzac139_Supplemental_File [file nzac139_supplemental_file.docx]

**Supplemental tables for:** The relationship between ferritin and body mass index is mediated by inflammation among women in higher-income countries, but not in most lower-income countries nor among young children: a multi-country analysis

**Authors:** Jennie N. Davis, Anne Williams, Charles D. Arnold, Fabian Rohner, James P. Wirth, Yaw Addo, Rafael C. Flores-Ayala, Brietta M. Oaks, Parminder S. Suchdev, Melissa F. Young, Reina Engle-Stone

| **Table of Contents for Supplemental Tables** | | |
| --- | --- | --- |
| **Table No.** | **Title** | **Page No.** |
| Supplemental Table 1 | Number and proportion of observations excluded due to exclusion criteria for WRA, by survey: BRINDA project | 3 |
| Supplemental Table 2 | Number and proportion of observations excluded due to exclusion criteria for PSC, by survey: BRINDA project | 5 |
| Supplemental Table 3 | Available covariates in each survey: BRINDA project | 7 |
| Supplemental Table 4 | Age and household characteristics for women of reproductive age (15-49 years) with normal weight to overweight/obesity by survey: BRINDA project | 8 |
| Supplemental Table 5 | Age, sex, and household characteristics for preschool-age children (6-59 months) with normal weight to overweight/obesity by survey: BRINDA project | 9 |
| Supplemental Table 6 | Bivariate and multivariable percent change associations between ferritin, CRP, AGP, and BMI among women of reproductive age (15-49 years) with normal weight to overweight/obesity by survey: BRINDA project | 11 |
| Supplemental Table 7 | Unadjusted and adjusted associations between ferritin, CRP or AGP, and BMI stratified by age among women of reproductive age (15-49 years) with normal weight to overweight/obesity by survey: BRINDA project | 13 |
| Supplemental Table 8 | Bivariate and multivariable percent change associations between ferritin, CRP, AGP, and BAZ among preschool-age children (2-5 years) with normal weight to overweight/obesity by survey: BRINDA project | 14 |
| Supplemental Table 9 | Unadjusted and adjusted associations between ferritin, CRP or AGP, and BAZ stratified by age among preschool-age children (6-59 months) with normal weight to overweight/obesity by survey: BRINDA project | 16 |
| Supplemental Table 10 | Unadjusted and adjusted associations between ferritin, CRP or AGP, and BAZ stratified by sex among preschool age children (6-59 months) with normal weight to overweight/obesity by survey: BRINDA project | 18 |
| Supplemental Table 11 | Unadjusted relationships between ferritin and BMI or BAZ as mediated by inflammation among women of reproductive age (15-49 years) and preschool-age children (6-59 months) with normal weight to overweight/obesity by survey: BRINDA project | 20 |
| Supplemental Table 12 | Sensitivity analysis: mediation analysis (unadjusted) assessing the relationship between ferritin, BMI or BAZ and inflammation including and excluding observations that tested positive for malaria for WRA and PSC with normal weight to overweight/obesity: BRINDA project | 22 |
|  | Supplemental References | 23 |

**Supplemental Table 1.** Number and proportion of observations excluded due to exclusion criteria for WRA, by survey: BRINDA project^1^

|  |  | **Exclusion Criteria^2^** | | | | | | | | |  |  |
| --- | --- | --- | --- | --- | --- | --- | --- | --- | --- | --- | --- | --- |
| **Survey, year** | **Total observations in dataset** | **BMI <18.5 kg/m^2^** | **Implausible Height** | **Implausible Weight** | **Pregnant** | **Positive Malaria Result** | **Missing BMI** | **Missing SF** | **Missing CRP** | **Missing AGP** | **Total observations excluded** | **Analytical sample size & proportion of total** |
|  | n | n (%) | n (%) | n (%) | n (%) | n (%) | n (%) | n (%) | n (%) | n (%) | n (%) | n (%) |
| Afghanistan, 2013 | 23875 | 954 (4.0) | 0 (0) | 0 (0) | 0 (0) | n/a | 13991 (58.6) | 22825 (95.6) | 22825 (95.6) | 22825 (95.6) | 23304 (97.6) | 571 (2.4) |
| Burkina Faso, 2010 | 484 | 60 (12.4) | 0 (0) | 0 (0) | 0 (0) | 36 (7.4) | 70 (14.5) | 355 (73.3) | 355 (73.3) | 355 (73.3) | 423 (87.4) | 61 (12.6) |
| Cambodia, 2014 | 724 | 94 (13.0) | 2 (0.3) | 1 (0.1) | 0 (0) | n/a | 0 (0) | 19 (2.6) | 19 (2.6) | 19 (2.6) | 115 (15.9) | 609 (84.1) |
| Côte D'Ivoire, 2007 | 863 | 81 (9.4) | 11 (1.3) | 2 (0.2) | n/a | 39 (4.5) | 14 (1.6) | 29 (3.4) | 29 (3.4) | 29 (3.4) | 157 (18.2) | 706 (81.1) |
| Laos, 2006 | 863 | 123 (14.3) | 0 (0) | 0 (0) | n/a | n/a | 36 (4.2) | 47 (5.5) | 47 (5.5) | 47 (5.5) | 173 (20.1) | 690 (79.9) |
| Malawi, 2016 | 804 | 70 (8.7) | 1 (0.1) | 0 (0) | 0 (0) | 116 (14.4) | 17 (2.1) | 28 (3.5) | 28 (3.5) | 28 (3.5 | 210 (26.1) | 594 (73.9) |
| Cameroon, 2009 | 787 | 67 (8.5) | 0 (0) | 0 (0) | n/a | 108 (13.7) | 4 (0.5) | 27 (3.4) | 27 (3.4) | 27 (3.4) | 193 (24.5) | 594 (75.5) |
| Georgia, 2009 | 1846 | 86 (4.7) | 0 (0) | 0 (0) | n/a | n/a | 18 (1.0) | 158 (8.6) | 158 (8.6) | n/a | 241 (13.1) | 1605 (86.9) |
| India, 2011 | 972 | 529 (54.4) | 2 (0.2) | 0 (0) | 0 (0) | n/a | 6 (0.6) | 645 (66.4) | 647 (66.6) | 647 (66.6) | 825 (84.9) | 147 (15.1) |
| Nigeria, 2012 | 620 | 55 (8.9) | 0 (0) | 1 (0.2) | n/a | 56 (9.0) | 10 (1.6) | 0 (0) | 0 (0) | 0 (0) | 114 (18.4) | 506 (81.6) |
| Pakistan, 2011 | 22278 | 3024 (15.6) | 3 (0.01) | 4 (0.02) | 0 (0) | n/a | 361 (1.6) | 14186 (63.7) | 14381 (64.6) | 14017 (63.0) | 17274 (77.5) | 5004 (22.5) |
| Vietnam, 2010 | 1492 | 305 (20.4) | 0 (0) | 0 (0) | n/a | n/a | 1 (0.1) | 4 (0.3) | 9 (0.6) | n/a | 314 (21.1) | 1178 (78.9) |
| Azerbaijan, 2013 | 2910 | 138 (4.7) | 0 (0) | 0 (0) | 0 (0) | n/a | 73 (2.5) | 254 (8.7) | 254 (8.5) | 254 (8.7) | 382 (13.1) | 2528 (86.9) |
| Colombia, 2010 | 9697 | 573 (5.9) | 0 (0) | 0 (0) | n/a | n/a | 281 (2.9) | 614 (6.3) | 614 (6.3) | n/a | 1397 (14.4) | 8300 (85.6) |
| Mexico, 2006 | 3050 | 88 (2.9) | 0 (0) | 1 (0.03) | n/a | n/a | 22 (0.7) | 26 (0.9) | 18 (0.6) | n/a | 140 (4.6) | 2910 (95.4) |
| Mexico, 2012 | 4176 | 97 (2.3) | 0 (0) | 0 (0) | n/a | n/a | 32 (0.8) | 564 (13.5) | 545 (13.1) | n/a | 636 (15.2) | 3540 (84.8) |
| United Kingdom, 2014 | 2050 | 69 (3.7) | 0 (0) | 0 (0) | 0 (0) | n/a | 93 (4.5) | 1123 (54.8) | 1108 (54.1) | n/a | 1188 (58.0) | 862 (42.0) |
| United States, 2006 | 3456 | 143 (4.1) | 0 (0) | 1 (0.03) | 0 (0) | n/a | 47 (1.4) | 273 (7.9) | 259 (7.5) | n/a | 432 (12.5) | 3024 (87.5) |

^1^Criteria for exclusion from analyses were: BMI <18.5 kg/m^2^; height or weight outside the ranges of 101.6-219.9 cm and 22.7-222.2 kg;(1) pregnancy; positive test result for malaria; or missing values for ferritin, CRP, AGP, or BMI (due to missing values for weight or height). Some observations may be excluded from multiple categories. Abbreviations: α, alpha-1-glycoprotein; BRINDA, Biomarkers Reflecting Nutritional Determinants of Anemia; CRP, C-reactive protein; WRA, women of reproductive age (15-49 years).

^2^Exclusion criteria percentages are proportions of total observations in the individual datasets.

**Supplemental Table 2.** Number and proportion of observations excluded due to exclusion criteria for PSC, by survey: BRINDA project^1^

|  |  | **Exclusion Criteria^2^** | | | | | | | | |  |  |
| --- | --- | --- | --- | --- | --- | --- | --- | --- | --- | --- | --- | --- |
| **Survey, year** | **Total observations in dataset** | **BAZ**  **<-2 SD** | **WHZ <-2 SD** | **Implausible BAZ** | **Implausible WHZ** | **Positive Malaria Result** | **Missing BAZ** | **Missing SF** | **Missing CRP** | **Missing AGP** | **Total observations excluded** | **Analytical sample size & proportion of total** |
|  | n | n (%) | n (%) | n (%) | n (%) | n (%) | n (%) | n (%) | n (%) | n (%) | n (%) | n (%) |
| Afghanistan, 2013 | 19896 | 1598 (8.0) | 1823 (9.2) | 744 (3.8) | 424 (2.1) | n/a | 341 (1.7) | 19231 (96.7) | 19231 (96.7) | 19231 (96.7) | 19301 (97.0) | 595 (3.0) |
| Bangladesh, 2010 | 1561 | 305 (19.5) | 277 (17.7) | 12 (0.8) | 8 (0.5) | n/a | 18 (1.2) | 68 (4.4) | 68 (4.4) | 68 (4.4) | 382 (24.5) | 1179 (75.5) |
| Bangladesh, 2012 | 1108 | 134 (12.1) | 161 (14.5) | 26 (2.3) | 19 (1.7) | n/a | 85 (7.7) | 640 (57.8) | 637 (57.5) | 637 (57.5) | 740 (33.2) | 368 (33.2) |
| Burkina Faso, 2010 | 482 | 5 (1.1) | 8 (1.7) | 0 (0) | 0 (0) | 9 (1.9) | 73 (15.1) | 357 (74.1) | 357 (74.1) | 357 (74.1) | 419 (86.9) | 63 (13.1) |
| Cambodia, 2014 | 874 | 60 (6.9) | 69 (7.9) | 5 (0.6) | 1 (0.1) | n/a | 0 (0) | 209 (23.9) | 209 (23.9) | 209 (23.9) | 275 (31.5) | 599 (68.5) |
| Côte D'Ivoire, 2007 | 864 | 104 (12.0) | 116 (13.4) | 29 (3.4) | 16 (1.9) | 214 (24.8) | 37 (4.3) | 118 (13.7) | 118 (13.7) | 118 (13.7) | 429 (49.7) | 435 (50.3) |
| Kenya, 2007 | 1056 | 47 (4.5) | 51 (4.8) | 5 (0.5) | 5 (0.5) | 196 (18.6) | 40 (3.8) | 160 (15.2) | 160 (15.2) | 160 (15.2) | 391 (37.0) | 665 (63.0) |
| Kenya, 2010 | 896 | 26 (2.9) | 29 (3.2) | 4 (0.5) | 3 (0.3) | 276 (30.8) | 31 (3.5) | 47 (5.3) | 47 (5.3) | 47 (5.3) | 345 (38.5) | 551 (61.5) |
| Laos, 2006 | 514 | 25 (4.9) | 40 (7.8) | 1 (0.2) | 1 (0.2) | n/a | 8 (1.6) | 32 (6.2) | 32 (6.2) | 33 (6.4) | 71 (13.8) | 443 (86.2) |
| Liberia, 2011 | 1476 | 129 (8.7) | 152 (10.3) | 3 (0.2) | 3 (0.2) | 358 (24.3) | 10 (0.7) | 42 (2.9) | 42 (2.9) | 42 (2.9) | 520 (35.2) | 956 (64.8) |
| Malawi, 2016 | 1233 | 48 (3.9) | 52 (4.2) | 12 (1.0) | 8 (0.7) | 310 (25.1) | 24 (2.0) | 131 (10.6) | 131 (10.6) | 131 (10.6) | 485 (39.3) | 748 (60.7) |
| Mongolia, 2006 | 242 | 0 (0) | 0 (0) | 0 (0) | 0 (0) | n/a | 1 (0.4) | 2 (0.8) | n/a | 2 (0.8) | 239 (1.2) | 239 (98.8) |
| Nicaragua, 2005 | 1424 | 11 (0.8) | 10 (0.8) | 2 (0.1) | 2 (0.1) | n/a | 4 (0.3) | 467 (32.8) | n/a | 0 (0) | 478 (33.6) | 946 (66.4) |
| Zambia, 2009 | 885 | 5 (0.6) | 6 (0.7) | 1 (0.1) | 0 (0) | n/a | 2 (0.2) | 473 (53.5) | 473 (53.4) | 474 (53.4) | 555 (62.7) | 330 (37.3) |
| Cameroon, 2009 | 853 | 16 (1.9) | 29 (3.4) | 1 (0.1) | 0 (0) | 195 | 22.9 | 61 (7.2) | 61 (7.2) | 61 (.7.2 | 297 (34.8) | 556 (65.2) |
| Georgia, 2009 | 2489 | 31 (1.2) | 20 (0.8) | 57 (2.3) | 43 (1.7) | n/a | 103 (4.1) | 347 (13.9) | 347 (13.9) | n/a | 425 (17.1) | 2064 (82.9) |
| Nigeria, 2012 | 640 | 61 (9.5) | 63 (9.8) | 30 (4.7) | 25 (3.9) | 198 (30.9) | 13 (2.0) | 93 (14.5) | 93 (14.5) | 93. (14.5) | 337 (52.7) | 303 (47.3) |
| Pakistan, 2011 | 10689 | 1365 (12.8) | 1543 (14.4) | 361 (3.4) | 280 (2.6) | n/a | 524 (4.9) | 3467 (32.4) | n/a | 3132 (29.3) | 4865 (45.5) | 5824 (54.5) |
| Philippines, 2011 | 1784 | 80 (4.5) | 98 (5.5) | 6 (0.3) | 4 (0.2) | n/a | 6 (0.3) | 17 (1.0) | 17 (1.0) | 17 (1.0) | 128 (7.2) | 1656 (92.8) |
| Vietnam, 2010 | 395 | 23 (5.8) | 25 (6.3) | 2 (0.5) | 1 (0.2) | n/a | 3 (0.8) | 15 (3.8) | 17 (4.3) | n/a | 51 (12.9) | 344 (87.1) |
| Azerbaijan, 2013 | 1404 | 41 (2.9) | 39 (2.8) | 49 (3.5) | 39 (2.8) | n/a | 49 (3.5 | 351 (25.0) | 351 (25.0) | 351 (25.0) | 417 (29.7) | 987 (70.3) |
| Colombia, 2010 | 7753 | 66 (0.9) | 76 (1.0) | 11 (0.1) | 7 (0.1) | n/a | 161 (2.1) | 3453 (44.5) | 3887 (50.1) | n/a | 3973 (51.2) | 3780 (48.8) |
| Mexico, 2006 | 6618 | 93 (1.4) | 90 (1.4) | 10 (0.2) | 6 (0.1) | n/a | 538 (8.1) | 5028 (76.0) | 5026 (76.0) | n/a | 5056 (76.4) | 1562 (23.6) |
| Mexico, 2012 | 8528 | 101 (1.2) | 102 (1.2) | 0 (0) | 0 (0) | n/a | 482 (5.7) | 5904 (69.2) | 5989 (70.2) | n/a | 6074 (71.2) | 2454 (28.8) |
| United States, 2006 | 2665 | 21 (0.8) | 20 (0.8) | 11 (0.4) | 7 (0.3) | n/a | 131 (4.9) | 1512 (56.8) | 1350 (50.7) | n/a | 1584 (59.4) | 1081 (40.6) |

^1^Criteria for exclusion from analyses were: BAZ or WHZ <-2 SD; BAZ or WHZ less than -5 SD or greater than 5 SD;(2) positive test result for malaria; or missing values for ferritin, CRP, AGP, or BAZ (due to missing values for weight or height/length). Some observations may be excluded from multiple categories. Abbreviations: α, alpha-1-glycoprotein; BAZ, BMI-for-age z-score; BRINDA, Biomarkers Reflecting Nutritional Determinants of Anemia; CRP, C-reactive protein; PSC, preschool-age children (6-59 months); WHZ, weight-for-height z-score.

^2^Exclusion criteria percentages are proportions of total observations in the individual datasets.

**Supplemental Table 3.** Available covariates in each survey: BRINDA project^1^

| **Survey** | **Age** | **Rural/Urban Setting** | **SES** | **Water** | **Sanitation** | **Respondent/Maternal**  **Education Level** | **Household Head Education Level** |
| --- | --- | --- | --- | --- | --- | --- | --- |
| Afghanistan | PSC, WRA | -- | PSC, WRA | PSC, WRA | PSC, WRA | PSC, WRA | -- |
| Azerbaijan | PSC, WRA | PSC, WRA | PSC, WRA | PSC, WRA | PSC, WRA | WRA only | -- |
| Bangladesh, 2010 | PSC, WRA | -- | -- | PSC only | PSC only | -- | -- |
| Bangladesh, 2012 | PSC, WRA | PSC only | PSC only | PSC only | PSC only | PSC only | -- |
| Burkina Faso | PSC, WRA | -- | PSC, WRA | PSC, WRA | PSC, WRA | -- | PSC, WRA |
| Cambodia | PSC, WRA | PSC, WRA | PSC, WRA | PSC, WRA | PSC, WRA | PSC, WRA | -- |
| Cameroon | PSC, WRA | PSC, WRA | PSC, WRA | PSC, WRA | PSC, WRA | PSC, WRA | -- |
| Colombia | PSC, WRA | PSC, WRA | PSC, WRA | PSC, WRA | PSC, WRA | WRA only | PSC only |
| Côte d’Ivoire | PSC, WRA | PSC, WRA | PSC, WRA | WRA only | PSC, WRA | PSC, WRA | -- |
| Georgia | PSC, WRA | PSC, WRA | -- | -- | -- | WRA only | -- |
| India | PSC, WRA | WRA only | -- | WRA only | WRA only | WRA only | -- |
| Kenya, 2007 | PSC, WRA | PSC only | PSC only | PSC only | PSC only | PSC only | -- |
| Kenya, 2010 | PSC, WRA | PSC only | PSC only | PSC only | PSC only | PSC only | -- |
| Laos | PSC, WRA | PSC, WRA | PSC, WRA | PSC, WRA | PSC, WRA | PSC, WRA | -- |
| Liberia | PSC, WRA | PSC only | PSC only | PSC only | PSC only | -- | -- |
| Malawi | PSC, WRA | PSC, WRA | PSC, WRA | PSC, WRA | PSC, WRA | PSC, WRA | -- |
| Mexico, 2006 | PSC, WRA | PSC, WRA | PSC, WRA | -- | PSC, WRA | -- | PSC, WRA |
| Mexico, 2012 | PSC, WRA | PSC, WRA | PSC, WRA | -- | -- | -- | -- |
| Mongolia | PSC, WRA | PSC only | -- | -- | -- | PSC only | -- |
| Nicaragua | PSC, WRA | PSC only | -- | PSC only | PSC only | PSC only | -- |
| Nigeria | PSC, WRA | PSC, WRA | -- | -- | -- | -- | -- |
| Pakistan | PSC, WRA | PSC, WRA | PSC, WRA | PSC, WRA | PSC, WRA | PSC, WRA | -- |
| Philippines | PSC, WRA | PSC only | PSC only | -- | PSC only | PSC only | -- |
| United Kingdom | PSC, WRA | -- | WRA only | -- | -- | WRA only | -- |
| United States | PSC, WRA | -- | PSC, WRA | -- | -- | WRA only | PSC only |
| Vietnam | PSC, WRA | PSC, WRA | -- | -- | -- | -- | -- |
| Zambia | PSC, WRA | PSC only | -- | -- | -- | -- | -- |

^1^Covariate definitions: age in years (WRA) or months (PSC); sex (male/female; PSC only); urban or rural residence; household socioeconomic status (SES) categorized as low SES versus high SES (variable created from the ordinal 3-category SES variable from the harmonized BRINDA dataset (3), which was then dichotomized into a binary variable of low SES versus high SES, where high SES included both medium and high categories); water defined as access to an improved water source (compared to no access or access only to an unimproved water source); sanitation defined as access to an improved toilet (compared to no access or access only to an unimproved toilet); and education level defined as either none or primary school only versus secondary school or more and measured as either the respondent (WRA) or maternal (PSC) education level, except in surveys that reported household head education level. BRINDA: Biomarkers Reflecting Inflammation and Nutritional Determinants of Anemia; PSC, preschool-age children (6-59 months); SES, socio-economic status; WRA, women of reproductive age (15-49 years).

**Supplemental Table 4.** Age and household characteristics for women of reproductive age (15-49 years) with normal weight to overweight/obesity by survey: BRINDA project^1^

| **Country Income Class-**  **ification^2^** | **Survey, year** | **n** | **Age, yr**  **median [IQR]** | **Urban residence**  **% (95% CI)** | **High SES^3^**  **% (95% CI)** | **Improved Water Source^4^**  **% (95% CI)** | **Improved Toilet^5^**  **% (95% CI)** | **High Education^6^**  **% (95% CI)** |
| --- | --- | --- | --- | --- | --- | --- | --- | --- |
| Low | Afghanistan, 2013 | 571 | 29.2 [24.3, 29.8] | -- | 93.1 (89.6, 96.6) | 81.5 (75.7, 87.4) | 56.6 (47.8, 65.3) | 10.2 (6.1, 14.4) |
|  | Burkina Faso, 2010 | 61 | 30.2 [25.3, 37.7] | -- | 76.5 (57.9, 95) | 31.3 (5.8, 56.7) | 13.0 (0.9, 25.1) | 2.1 (0.0, 7.6) |
|  | Cambodia, 2014 | 609 | 29.8 [25.1, 33.6] | 13.1 (9.9, 16.3) | 60.5 (53.7, 67.3) | 53.1 (45.7, 60.6) | 52.1 (46.6, 57.7) | 31.7 (26.5, 36.9) |
|  | Côte d’Ivoire, 2007 | 706 | 26.3 [21.1, 31.9] | 56.1 (51.9, 60.3) | 63.9 (58.1, 69.8) | 88.0 (82.6, 93.3) | 89.9 (85.9, 93.8) | 15.5 (11.5, 19.4) |
|  | Laos, 2006 | 690 | 28.5 [20.4, 37.2] | 33.1 (19.2, 46.9) | 61.9 (51.2, 72.5) | 42.4 (33.5, 51.3) | 90.6 (84.1, 97.1) | 34.8 (25.4, 44.1) |
|  | Malawi, 2016 | 594 | 27.8 [20.8, 36.8] | 10.3 (1.9, 18.7) | 61.0 (52.9, 69.0) | 83.6 (75.7, 91.4) | 83.8 (76.4, 91.2) | 22.1 (15.7, 28.5) |
| Low-middle | Cameroon, 2009 | 594 | 26.2 [22.0, 31.8] | 62.8 (52.5, 73.2) | 64.1 (56.7, 71.6) | 75.0 (68.9, 81.0) | 67.0 (61.5, 72.6) | 37.5 (33.3, 41.7) |
|  | Georgia, 2009 | 1605 | 32.2 [24.2, 41.0] | 50.3 (43.2, 57.3) | -- | -- | -- | 95.3 (93.8, 96.8) |
|  | India, 2011 | 147 | 25.2 [21.6, 31.2] | 0^7^ | -- | 90.5 (83.1, 97.9) | 23.1 (12.6, 33.6) | 59.9 (42.8, 77.0) |
|  | Nigeria, 2012 | 506 | 26.3 [21.5, 31.1] | 0^7^ | -- | -- | -- | -- |
|  | Pakistan, 2011 | 5004 | 29.6 [25.8, 34.6] | 32.0 (28.8, 35.3) | 61.2 (58.6, 63.8) | 93.0 (91.7, 94.4) | 84.5 (82.7, 86.4) | 30.3 (28.0, 32.5) |
|  | Vietnam, 2010 | 1178 | 33.4 [25.9, 40.9] | 50.5 (48.2, 52.8) | -- | -- | -- | -- |
| Upper-middle | Azerbaijan, 2013 | 2528 | 31.7 [23.9, 41.4] | 45.7 (39.4, 51.9) | 68.6 (65.1, 72.2) | 77.1 (72.2, 82.1) | 93.7 (90.9, 96.5) | 95.0 (93.5, 96.5) |
|  | Colombia, 2010 | 8300 | 28.6 [19.4, 39.2] | 77.5 (76.5, 78.6) | 62.0 (60.6, 63.4) | 87.0 (85.4, 88.6) | 97.2 (96.3, 98.0) | 49.1 (46.9, 51.3) |
|  | Mexico, 2006 | 2910 | 31.6 [23.0, 40.1] | 71.3 (67.0, 75.6) | 53.0 (49.1, 57.0) | -- | 87.9 (85.3, 90.5) | 41.6 (38.0, 45.3) |
|  | Mexico, 2012 | 3540 | 33.5 [27.4, 40.6] | 79.2 (77.4, 80.9) | 69.8 (67.0, 72.6) | -- | -- | -- |
| High | United Kingdom, 2014 | 862 | 33.4 [23.3, 41.9] | -- | 63.0 (57.9, 68.2) | -- | -- | 91.4 (88.7, 94.1) |
|  | United States, 2006 | 3024 | 34.7 [24.7, 42.5] | -- | 74.2 (71.0, 77.4) | - | -- | 100.0 (100,100) |

^1^Estimates account for complex survey design (cluster, strata) with survey weights applied. Inclusion criteria were: BMI ≥18.5 kg/m^2^, not pregnant, and a negative malaria test result. ‘--‘ indicates the variable was unavailable in that survey. BMI, body mass index; BRINDA, Biomarkers Reflecting Inflammation and Nutritional Determinants of Anemia; SES, socioeconomic status.

^2^Country income classification defined according to the World Bank definition for the year the survey took place (4).

^3^A binary SES variable (low/high where high includes both medium and high categories) was created from the 3-level ordinal SES variable available from the harmonized BRINDA dataset,(3) which was created from survey-specific asset scores (quintiles) of household ownership or composition.

^4^‘Improved Water Source’ (compared to no access or access only to an unimproved water source) was defined as having access to: piped water in a dwelling/yard; a communal/public tap; a borehole/tube well, owned or shared; a protected well/spring; a protected open dug well; or rain water.

^5^‘Improved Toilet’ (compared to no access or access only to an unimproved toilet) was defined as have access to: a flush toilet/pit latrine flush to piped sewer; a ventilated improved pit/latrine/Sanplat; or a flush to pit/latrine.

^6^‘High Education’ (compared to none or primary school only) was measured as respondent education level or head of household education level in surveys in which respondent education was not measured (Burkina Faso and Mexico 2006).

^7^Surveys from India and Nigeria contained only observations from rural areas.

**Supplemental Table 5.** Age, sex, and household characteristics for preschool-age children (6-59 months) with normal weight to overweight/obesity by survey: BRINDA project^1^

| **Country Income Class-**  **ification^2^** | **Country, survey year** | **n** | **Age, mo**  **median [IQR]** | **Male**  **%** | **Urban residence**  **% (95% CI)** | **High SES^3^**  **% (95% CI)** | **Improved Water Source^4^**  **% (95% CI)** | **Improved Toilet^5^**  **% (95% CI)** | **High Education^6^**  **% (95% CI)** |
| --- | --- | --- | --- | --- | --- | --- | --- | --- | --- |
| Low | Afghanistan, 2013 | 595 | 27.8 [17.0, 39.9] | 51.9 | -- | 94.9 (92.5, 97.3) | 81.5 (74.1, 89.0) | 68.1 (60.1, 76.0) | 9.8 (5.3, 14.4) |
|  | Bangladesh, 2010 | 1179 | 7.7 [6.3, 9.3] | 49.1 | -- | -- | 98.4 (95.3, 100.0) | 25.2 (16.9, 33.5) | -- |
|  | Bangladesh, 2012 | 368 | 37.9 [26.8, 48.0] | 54.6 | 26.9 (19.1, 34.7) | 44.3 (33.1, 55.5) | 99.0 (97.5, 100.0) | 63.3 (49.5, 77.1) | 47.1 (36.0, 58.2) |
|  | Burkina Faso, 2010 | 63 | 48.3 [43.6, 53.5] | 44.4 | -- | 80.1 (63.3, 96.8) | 29.6 )8.5, 50.6) | 12.2 (0, 27.7) | 2.1 (0, 7.1) |
|  | Cambodia, 2014 | 599 | 37.0 [24.6, 48.3] | 54.4 | 11.6 (8.5, 14.7) | 58.4 (50.0, 66.7) | 53.4 (44.4, 62.3) | 53.9 (46.7, 61.1) | 27.7 (22.5, 32.9) |
|  | Côte d’Ivoire, 2007 | 435 | 29.6 [17.3, 44.2] | 54.7 | 58.2 (52.0, 64.5) | 67.7 (62.1, 73.4) | -- | 66.6 (58.0, 75.3) | 12.4 (9.3, 15.4) |
|  | Kenya, 2007 | 665 | 18.6 [12.1, 26.4] | 51.1 | 0^7^ | 60.0 (54.3, 65.8) | 52.6 (42.9, 62.4) | 0.2 (0.0, 0.5) | 14.7 (11.7, 17.7) |
|  | Kenya, 2010 | 551 | 21.7 [13.4, 27.0] | 50.5 | 0^7^ | 61.7 (56.1, 67.2) | 58.2 (49.0, 67.3) | 1.1 (0.1, 2.2) | 16.4 (12.5, 20.3) |
|  | Laos, 2006 | 443 | 33.4 [20.7, 45.6] | 49.4 | 14.3 (5.7, 22.8) | 38.9 (28.7, 49.2) | 43.9 (32.3, 55.5) | 28.5 (17.9, 39.1) | 15.8 (8.1, 23.6) |
|  | Liberia, 2011 | 956 | 17.3 [11.2, 26.0] | 47.6 | 43.6 (39.0, 48.2) | 68.3 (61.6, 75.0) | 83.2 (61.6, 75.0) | 43.5 (37.0, 50.0) | -- |
|  | Malawi, 2016 | 748 | 31.4 [18.5, 44.7] | 51.3 | 13.7 (0, 28.0) | 56.5 (49.6, 63.3) | 87.2 (81.0, 93.4) | 82.6 (77.5, 87.6) | 24.0 (13.1, 34.9) |
|  | Mongolia, 2006 | 239 | 18.9 [12.3, 26.7] | 51.9 | 49.0 (42.5, 55.5) | -- | -- | -- | 80.3 (74.7, 85.2) |
|  | Nicaragua, 2005 | 946 | 34.0 [19.0, 47.0] | 50.1 | 62.2 (51.0, 73.3) | -- | 89.1 (82.9, 95.2) | 36.4 (28.9, 43.9) | 45.8 (37.4, 54.2) |
|  | Zambia, 2009 | 330 | 37.0 [23.9, 48.0] | 57.6 | 21.8 (15.9, 27.7) | -- | -- | -- | -- |
| Low-middle | Cameroon, 2009 | 556 | 29.0 [18.8, 38.9] | 50.7 | 61.8 (51.5, 72.1) | 65.8 (57.9, 73.7) | 77.4 (71.4, 83.3) | 67.2 (61.3, 73.1) | 37.7 (32.8, 42.6) |
|  | Georgia, 2009 | 2064 | 36.3 [23.5, 50.2] | 53.7 | 47.3 (40.5, 54.0) | -- | -- | -- | -- |
|  | Nigeria, 2012 | 303 | 29.5 [20.9, 35.7] | 50.5 | 0^7^ | -- | -- | -- | -- |
|  | Pakistan, 2011 | 5824 | 25.6 [14.8, 39.3] | 51.2 | 30.9 (27.8, 34.0) | 58.8 (56.2, 61.4) | 94.9 (93.7, 96.0) | 91.6 (90.4, 92.7) | 20.5 (18.7, 22.4) |
|  | Philippines, 2011 | 1656 | 15.4 [10.7, 19.0] | 49.5 | 9.1 (8.4, 9.8) | 16.0 (12.8, 19.1) | 44.6 (40.0, 49.3) | 92.6 (87.5, 97.7) | 66.6 (61.3, 71.9) |
|  | Vietnam, 2010 | 344 | 37.5 [25.9, 49.2] | 52.3 | 47.4 (42.0, 52.8) | -- | -- | -- | -- |
| Upper-middle | Azerbaijan, 2013 | 987 | 36.5 [23.6, 47.1] | 55.0 | 45.3 (38.1, 52.5) | 68.6 (64.1, 73.1) | 77.4 (71.8, 83.0) | 93.0 (89.9, 96.2) | -- |
|  | Colombia, 2010 | 3780 | 37.6 [25.7, 49.0] | 52.7 | 69.9 (68.6, 71.2) | 48.6 (46.5, 50.7) | 86.6 (83.8, 89.5) | 88.5 (86.2, 90.9) | 42.8 (39.8, 45.8) |
|  | Mexico, 2006 | 1562 | 43.4 [32.6, 52.5] | 52.4 | 52.0 (47.7, 56.3) | 34.6 (30.6, 38.6) | -- | 74.4 (70.4, 78.3) | 37.1 (33.2, 41.0) |
|  | Mexico, 2012 | 2454 | 37.0 [35.2, 38.8] | 49.3 | 72.2 (69.3, 75.2) | 53.5 (49.7, 57.3) | -- | -- | -- |
| High | USA, 2006 | 1081 | 40.0 [25.2, 50.0] | 50.6 | -- | 59.4 (54.1, 64.7) | -- | -- | 93.3 (91.4, 95.3) |

^1^Estimates account for the complex survey design (cluster, strata) with survey weights applied, except in the survey from Mongolia which followed a simple random sampling design. Inclusion criteria were: BMI-for-age z-score or weight-for-height z-score ≥-2 SD and a negative malaria test result. ‘--‘ indicates the variable was unavailable in that survey. BRINDA, Biomarkers Reflecting Inflammation and Nutritional Determinants of Anemia; SES, socioeconomic status.

^2^Country income classification defined according to the World Bank definition for the year the survey took place (4).

^3^A binary SES variable (low/high) was created from the 3-level ordinal SES variable available harmonized BRINDA dataset (3), which was created from survey-specific asset scores (quintiles) of household ownership or composition.

^4^‘Improved Water Source’ (compared to no access or access only to an unimproved water source) was defined as having access to: piped water in a dwelling/yard; a communal/public tap; a borehole/tube well, owned or shared; a protected well/spring; a protected open dug well; or rain water.

^5^‘Improved Toilet’ (compared to no access or access only to an unimproved toilet) was defined as have access to: a flush toilet/pit latrine flush to piped sewer; a ventilated improved pit/latrine/Sanplat; or a flush to pit/latrine.

^6^‘High Education’ (compared to none or primary school only) was measured as maternal education level or head of household education level in surveys in which maternal education was not measured (Burkina Faso, Colombia, Mexico 2006, and the United States).

^7^Surveys from Kenya (2007 and 2010) and Nigeria contained only observations from rural areas.

**Supplemental Table 6.** Bivariate and multivariable percent change associations between ferritin, CRP, AGP, and BMI among women of reproductive age (15-49 years) with normal weight to overweight/obesity by survey: BRINDA project^1^

|  | | | | **Bivariate (unadjusted) and multivariable (adjusted) linear regression** | | | | |
| --- | --- | --- | --- | --- | --- | --- | --- | --- |
| **Country Income Classification** | **Country, survey year** | **n** |  | **Ferritin**  **regressed on BMI** | **CRP**  **regressed on BMI** | **AGP**  **regressed on BMI** | **Ferritin**  **regressed on *ln*CRP** | **Ferritin**  **regressed on *ln*AGP** |
| Low-income | Afghanistan, 2013 | 571 | B | -2.1 (-4.3, 0.2) | 4.9 (-2.2, 12.5) | 0.6 (-0.2, 1.4) | 7.7 (2.3, 13.4) | 123.1 (54.1, 222.9) |
|  |  |  | M | -2.1 (-4.3, 0.2) | 4.4 (-2.7, 12.1) | 0.6 (-0.2, 1.4) | 7.7 (2.3, 13.4) | 123.1 (54.1, 222.9) |
|  | Burkina Faso, 2010 | 61 | B | -2.9 (-17.6, 14.4) | -21.8 (-34.3, -7.0) | -3.5 (-9.9, 3.3) | 29.9 (9.0, 54.7) | 118.0 (14.9, 313.6) |
|  |  |  | M | -1.3 (-13.9, 13.1) | -22.3 (-35.2, -6.9) | -4.0 (-9.8, 2.2) | 28.8 (12.3, 47.7) | 115.8 (9.9, 323.7) |
|  | Cambodia, 2014 | 609 | B | 3.7 (2.0, 5.5) | 17.1 (13.4, 20.9) | 2.5 (0.5, 4.6) | 14.7 (9.5, 20.1) | 43.0 (30.2, 57.2) |
|  |  |  | M | 3.4 (1.7, 5.1) | 16.7 (12.9, 20.5) | 2.9 (0.8, 5.0) | 13.9 (8.9, 19.2) | 42.9 (30.5, 56.6) |
|  | Côte d’Ivoire, 2007 | 706 | B | 0.2 (-1.4, 1.9) | 7.1 (4.7, 9.5) | 1.2 (0.6, 1.7) | 16.4 (10.5, 22.6) | 95.7 (57.2, 143.6) |
|  |  |  | M | 0.7 (-1.1, 2.4) | 6.8 (4.3, 9.2) | 1.2 (0.6, 1.7) | 16.9 (11.1, 23.0) | 97.4 (57.8, 146.8) |
|  | Laos, 2006 | 690 | B | 11.9 (8.6, 15.4) | 22.6 (18.4, 26.9) | 1.9 (1.3, 2.6) | 19.1 (12.5, 26.1) | 58.5 (6.3, 136.2) |
|  |  |  | M | 5.5 (2.4, 8.7) | 18.4 (14.2, 22.8) | 1.9 (1.3, 2.6) | 12.7 (7.5, 18.1) | 60.6 (20.1, 114.8) |
|  | Malawi, 2016 | 594 | B | -0.5 (-2.5, 1.6) | 10.8 (5.7, 16.1) | 2.9 (1.8, 4.1) | 2.6 (-2.2, 7.5) | 22.4 (5.8, 41.7) |
|  |  |  | M | -0.5 (-2.5, 1.6) | 10.7 (5.8, 15.9) | 3.2 (1.9, 4.4) | 2.6 (-2.2, 7.5) | 22.4 (5.8, 41.7) |
| Low-middle | Cameroon, 2009 | 594 | B | 0.0 (-1.3, 1.3) | 6.5 (3.7, 9.4) | 0.5 (0.2, 0.9) | 10.4 (5.3, 15.7) | 102.9 (50.3, 174.1) |
|  |  |  | M | 0.2 (-1.6, 1.2) | 6.8 (3.9, 9.9) | 0.6 (0.3, 1.0) | 8.6 (3.1, 14.4) | 82.4 (33.1, 149.8) |
|  | Georgia, 2009 | 1605 | B | 1.2 (0.7, 1.8) | 8.3 (6.6, 10.0) | -- | 6.1 (2.7, 9.6) | -- |
|  |  |  | M | 1.2 (0.7, 1.8) | 7.6 (5.7, 9.5) | -- | 6.1 (2.7, 9.6) | -- |
|  | India, 2011 | 147 | B | 7.5 (-0.4, 16.0) | 20.5 (6.4, 22.9) | 2.4 (1.2, 3.7) | 27.7 (13.4, 43.9) | 228.8 (65.7, 552.4) |
|  |  |  | M | 7.5 (-0.4, 16.0) | 13.6 (5.5, 22.4) | 2.4 (1.2, 3.7) | 27.7 (13.4, 43.9) | 228.8 (65.7, 552.4) |
|  | Nigeria, 2012 | 506 | B | -1.5 (-3.3, 0.4) | -0.6 (-3.5, 2.3) | 0.05 (-0.6, 0.7) | 13.3 (7.0, 20.1) | 111.2 (69.8, 162.6) |
|  |  |  | M | -1.5 (-3.3, 0.4) | -0.6 (-3.5, 2.3) | 0.05 (-0.6, 0.7) | 13.3 (7.0, 20.1) | 111.2 (69.8, 162.6) |
|  | Pakistan, 2011 | 5004 | B | 1.0 (0.4, 1.7) | 3.0 (2.0, 4.1) | 1.0 (0.7, 1.2) | 3.9 (1.5, 6.4) | 16.7 (7.3, 26.9) |
|  |  |  | M | 0.8 (0.1, 1.5) | 2.9 (1.8, 3.9) | 1.0 (0.7, 1.2) | 4.2 (1.8, 6.6) | 17.5 (7.6, 28.4) |
|  | Vietnam, 2010 | 1178 | B | 4.1 (1.6, 6.7) | 17.8 (15.1, 20.5) | -- | 20.8 (15.2, 26.7) | -- |
|  |  |  | M | 2.3 (-0.4, 5.0) | 17.1 (14.1, 20.1) | -- | 18.9 (13.4, 24.7) | -- |
| Upper-middle | Azerbaijan, 2013 | 2528 | B | 3.5 (2.7, 4.3) | 13.6 (12.3, 15.0) | 1.5 (1.3, 1.8) | 20.5 (16.8, 24.4) | 143.5 (102.3, 193.1) |
|  |  |  | M | 3.5 (2.5, 4.4) | 11.7 (10.3, 13.3) | 1.5 (1.2, 1.8) | 20.8 (16.8, 25.0) | 136.3 (95.1, 186.1) |
|  | Colombia, 2010 | 8300 | B | 2.0 (1.4, 2.6) | 13.1 (11.6, 14.6) | -- | 3.9 (2.7, 5.1) | -- |
|  |  |  | M | 2.1 (1.4, 2.7) | 13.0 (11.3, 14.8) | -- | 3.8 (2.6, 5.0) | -- |
|  | Mexico, 2006 | 2910 | B | 1.8 (0.8, 2.8) | 10.0 (8.2, 11.9) | -- | 13.2 (7.7, 19.0) | -- |
|  |  |  | M | 1.1 (0.02, 2.2) | 8.5 (6.6, 10.4) | -- | 10.2 (4.0, 16.7) | -- |
|  | Mexico, 2012 | 3540 | B | 2.7 (1.3, 4.2) | 11.4 (9.7, 13.2) | -- | 21.6 (15.7, 27.7) | -- |
|  |  |  | M | 2.7 (1.2, 4.2) | 11.4 (9.7, 13.1) | -- | 21.3 (15.5, 27.4) | -- |
| High | United Kingdom, 2014 | 862 | B | 1.9 (0.5, 3.3) | 6.5 (5.4, 7.6) | -- | 14.2 (4.8, 24.4) | -- |
|  |  |  | M | 1.6 (0.2, 3.1) | 6.5 (5.4, 7.6) | -- | 14.2 (4.7, 24.5) | -- |
|  | United States, 2006 | 3024 | B | 1.3 (0.6, 2.0) | 11.5 (10.9, 12.1) | -- | 12.9 (10.4, 15.4) | -- |
|  |  |  | M | 1.0 (0.3, 1.7) | 11.0 (10.4, 11.5) | -- | 11.0 (8.5, 13.5) | -- |

^1^Ferritin, CRP and AGP variables were *natural-log* transformed for analysis due to non-normal distributions. Regression estimates were exponentiated, and results are presented as the percent change (95% confidence interval) in the dependent variable for every 1-unit change in the independent variable. Note that for the values presented for ‘Ferritin regressed on *ln*CRP’ and ‘Ferritin regressed on *ln*AGP’, the percent changes in ferritin concentration are for every 1-unit change in *natural-log* transformed CRP or AGP, and the units differ (CRP, mg/L; AGP, g/L). See Table 2 of main manuscript for geometric mean CRP and AGP values by survey. All estimates account for the complex survey design (cluster, strata) with survey weights applied. Ferritin was measured in either serum or plasma, as reported by the survey. Covariates available for adjustment were: age, education level (respondent or household head), household socioeconomic status, access to an improved water source, access to an improved toilet, and urban/rural residence. Covariates were included in the multivariable regression model if they were associated with the outcome variable at p<0.1 in the bivariate model. Inclusion criteria were: BMI ≥18.5 kg/m^2^, not pregnant, and a negative malaria test result. Country income classification was defined according to the World Bank definition for the year in which the survey was conducted (4). ‘--‘ indicates the variable was unavailable in that survey. AGP, α-1-acid glycoprotein; B, bivariate model; BMI, body mass index; BRINDA, Biomarkers Reflecting Inflammation and Nutritional Determinants of Anemia; CRP, C-reactive protein; M, multivariable model.

**Supplemental Table 7.** Unadjusted and adjusted associations between ferritin, CRP or AGP, and BMI stratified by age among women of reproductive age (15-49 years) with normal weight to overweight/obesity by survey: BRINDA project^1^

|  |  |  | **Stratified analysis, unadjusted** | | **Stratified analysis, adjusted** | |
| --- | --- | --- | --- | --- | --- | --- |
|  |  |  | β (95% confidence interval) | | β (95% confidence interval) | |
| **Survey, year** | **n** | **Model** | **Ages 15-29 years** | **Ages 30-49 years** | **Ages 15-29 years** | **Ages 30-49 years** |
| Afghanistan, 2013 | 571 | No effect modification | **--** | **--** | **--** | **--** |
| Burkina Faso, 2010 | 61 | *ln*AGP=BMI | 0.06 (-0.09, 0.2) | -0.05 (-0.09, -0.02) | 0.05 (-0.09, 0.19) | -0.05 (-0.10, -0.02) |
| Cambodia, 2014 | 609 | No effect modification | **--** | **--** | **--** | **--** |
| Côte d’Ivoire, 2007 | 706 | *ln*Ferritin=*ln*CRP | 0.19 (0.13, 0.26) | 0.07 (-0.01, 0.15) | 0.19 (0.13, 0.26) | 0.08 (0.002, 0.16) |
| Laos, 2006 | 690 | *ln*Ferritin=BMI | 0.03 (-0.03, 0.1) | 0.13 (0.09, 0.16) | 0.05 (0.01, 0.1) | 0.09 (0.04, 0.13) |
|  |  | *ln*AGP=BMI | 0.01 (-0.001, 0.03) | 0.13 (0.09, 0.16) | no covariates to test | no covariates to test |
|  |  | *ln*Ferritin=*ln*CRP | 0.11 (0.04, 0.18) | 0.2 (0.12, 0.29) | 0.11 (0.05, 0.17) | 0.15 (0.07, 0.24) |
|  |  | *ln*Ferritin=*ln*AGP | 0.03 (-0.44, 0.5) | 0.93, 0.43, 1.43) | 0.19 (-0.19, 0.57) | 0.79, 0.37, 1.20) |
| Malawi, 2016 | 594 | No effect modification | **--** | **--** | **--** | **--** |
| Cameroon, 2009 | 594 | *ln*CRP=BMI | 0.04 (0.01, 0.08) | 0.09 (0.06, 0.13) | -0.01 (-0.03, 0.01) | 0.0006 (-0.02, 0.02) |
| Georgia, 2009 | 1605 | No effect modification | **--** | **--** | **--** | **--** |
| India, 2011 | 147 | *ln*Ferritin=*ln*CRP | 0.15 (-0.001, 03) | 0.37 (0.22, 0.53) | no covariates to test | no covariates to test |
|  |  | *ln*Ferritin=*ln*AGP | 0.6 (-0.14, 1.34) | 2.12 (1.38, 2.86) | no covariates to test | no covariates to test |
| Nigeria, 2012 | 506 | No effect modification | **--** | **--** | **--** | **--** |
| Pakistan, 2011 | 5004 | *ln*Ferritin=BMI | 0.02 (0.01, 0.03) | 0.01 (-0.004, 0.01) | 0.02 (0.01, 0.03) | 0.003 (-0.01, 0.01) |
|  |  | *ln*Ferritin=*ln*AGP | 0.24 (0.12, 0.36) | 0.1 (-0.01, 0.2) | 0.27 (0.14, 0.39) | 0.1 (-0.02, 0.21) |
| Vietnam, 2010 | 1178 | No effect modification | **--** | **--** | **--** | **--** |
| Azerbaijan, 2013 | 2528 | *ln*CRP=BMI | 0.17 (0.14, 0.19) | 0.1 (0.08, 0.11) | 0.16 (0.14, 0.18) | 0.1 (0.08, 0.11) |
|  |  | *ln*AGP=BMI | 0.02 (0.02, 0.03) | 0.01 (0.01, 0.02) | 0.02 (0.02, 0.03) | 0.01 (0.02, 0.03) |
| Colombia, 2010 | 8300 | No effect modification | **--** | **--** | **--** | **--** |
| Mexico, 2006 | 2910 | *ln*Ferritin=BMI | 0.005 (-0.02, 0.03) | 0.02 (0.002, 0.03) | no covariates to test | no covariates to test |
|  |  | *ln*CRP=BMI | 0.11 (0.08, 0.13) | 0.07 (0.05, 0.1) | no covariates to test | no covariates to test |
| Mexico, 2012 | 3450 | No effect modification | **--** | **--** | **--** | **--** |
| United Kingdom, 2014 | 862 | No effect modification | **--** | **--** | **--** | **--** |
| United States, 2006 | 3024 | No effect modification | **--** | **--** | **--** | **--** |

^1^Results presented as the unexponentiated β (95% confidence interval). Effect modification was evaluated through stratified analyses in each of the bivariate models listed above by testing for a significant interaction between the predictor (i.e., BMI, *ln*CRP, or *ln*AGP) and the effect modifier variable of age. Unadjusted stratified analyses were completed for all models where p<0.1 for the interaction. Adjusted stratified analyses were then completed for any marginally significant covariates (p<0.1) found in bivariate analyses. All estimates account for the complex survey design (cluster, strata) with survey weights applied. Covariates available for adjustment were: education level (respondent or household head), household socioeconomic status, access to an improved water source, access to an improved toilet, and urban/rural residence. Inclusion criteria were: BMI ≥18.5 kg/m^2^, not pregnant, and a negative malaria test result. AGP, α-1-acid glycoprotein; BMI; body mass index; BRINDA, Biomarkers Reflecting Inflammation and Nutritional Determinants of Anemia; CRP, C-reactive protein.

**Supplemental Table 8.** Bivariate and multivariable percent change associations between ferritin, CRP, AGP, and BAZ among preschool-age children (6-59 months) with normal weight to overweight/obesity by survey: BRINDA project^1^

|  |  |  |  | **Bivariate (unadjusted) and multivariable (adjusted) linear regression** | | | | |
| --- | --- | --- | --- | --- | --- | --- | --- | --- |
| **Country Income**  **Classification^3^** | **Country, survey year** | **n** |  | **Ferritin**  **regressed on BAZ** | **CRP**  **regressed on BAZ** | **AGP**  **regressed on BAZ** | **Ferritin**  **regressed on *ln*CRP** | **Ferritin**  **regressed on *ln*AGP** |
| Low | Afghanistan, 2013 | 595 | B | -1.4 (-10.2, 8.1) | -11.6 (-27.4, 7.5) | -3.9 (-6.1, -1.6) | 8.0 (3.6, 12.5) | 27.0 (-5.7, 71.1) |
|  |  |  | M | -2.8 (-0.4, 1.9) | -11.6 (-27.4, 7.5) | -3.9 (-6.1, -1.6) | 7.9 (3.6, 12.3) | 28.0 (-3.9, 70.4) |
|  | Bangladesh, 2010 | 1179 | B | -6.5 (-10.7, -2.0) | -3.8 (-11.9, 5.1) | -1.2 (-3.2, 0.9) | 10.9 (8.6, 13.3) | 113.9 (83.0, 149.9) |
|  |  |  | M | -6.7 (-11.0, -2.2) | -3.4 (-11.9, 5.8) | -0.9 (-2.9, 1.2) | 10.5 (8.2, 12.9) | 113.8 (81.5, 151.8) |
|  | Bangladesh, 2012 | 368 | B | -4.8 (-14.3, 5.7) | -4.7 (-14.8, 6.6) | -2.6 (-6.5, 1.5) | 27.6 (18.3, 37.5) | 148.5 (76.2, 250.3) |
|  |  |  | M | 2.9 (-6.7, 13.5) | -3.6 (-13.8, 7.8) | -1.8 (-5.3, 1.9) | 25.0 (17.4, 33.0) | 121.0 (64.1, 197.7) |
|  | Burkina Faso, 2010 | 63 | B | -10.5 (-38.5, 30.3) | -6.7 (-43.7, 54.8) | 0.1 (-9.2, 10.4) | 12.7 (-5.2, 34.0) | 184.4 (59.1, 408.4) |
|  |  |  | M | -9.8 (-35.7, 26.6) | -6.6 (-43.7, 54.9) | 0.3 (-8.1, 9.6) | 9.9 (-11.0, 35.8) | 173.0 (62.5, 358.4) |
|  | Cambodia, 2014 | 599 | B | -6.3 (-15.6, 4.0) | 10.5 (-3.4, 26.4) | 1.7 (-7.0, 11.1) | 16.2 (12.4, 20.1) | 62.2 (54.5, 70.4) |
|  |  |  | M | -3.7 (-11.3, 4.4) | 8.3 (-4.9, 23.4) | -- | 19.2 (15.2, 23.3) | 65.0 (57.3, 73.0) |
|  | Côte d’Ivoire, 2007 | 435 | B | 1.4 (-7.2, 10.7) | 8.1 (-7.5, 26.4) | -0.04 (-3.1, 3.2) | 31.9 (25.3, 39.0) | 132.9 (74.0, 211.7) |
|  |  |  | M | 2.9 (-5.5, 12.0) | 6.9 (-8.8, 25.3) | -1.1 (-4.2, 2.0) | 28.8 (22.6, 35.4) | 113.8 (57.8, 189.6) |
|  | Kenya, 2007 | 665 | B | -7.3 (-15.6, 1.8) | 4.5 (-7.6, 18.2) | -1.1 (-3.9, 1.7) | 19.9 (15.1, 24.8) | 135.9 (89.0, 194.5) |
|  |  |  | M | -6.1 (-14.4, 2.9) | 6.9 (-6.1, 21.7) | -1.0 (-3.9, 2.0) | 20.0 (15.1, 25.1) | 143.8 (94.2, 206.1) |
|  | Kenya, 2010 | 551 | B | -5.0 (-12.7, 3.4) | 7.7 (-9.5, 28.3) | 1.6 (-1.2, 4.5) | 25.2 (18.6, 32.1) | 324.2 (223.8, 455.8) |
|  |  |  | M | -6.1 (-13.7, 2.1) | 7.3 (-9.9, 27.9) | 1.3 (-1.6, 4.3) | 25.3 (18.7, 32.2) | 323.5 (224.5, 452.7) |
|  | Laos, 2006 | 443 | B | -5.8 (-16.8, 6.6) | -6.0 (-30.1, 26.5) | -2.0 (-6.7, 2.8) | 8.7 (2.7, 15.1) | 96.4 (29.1, 198.8) |
|  |  |  | M | -2.4 (-12.9, 9.3) | -5.3 (-27.0, 22.7) | -2.2 (-6.8, 2.6) | 12.1 (6.1, 18.5) | 133.6 (63.8, 233.0) |
|  | Liberia, 2011 | 956 | B | -1.6 (-6.8, 3.9) | 3.0 (-7.0, 14.1) | -0.6 (-2.6, 1.5) | 18.1 (12.7, 23.8) | 135.7 (74.2, 218.9) |
|  |  |  | M | -1.6 (-6.8, 3.9) | 1.8 (-8.0, 12.7) | -0.7 (-2.8, 1.4) | 18.1 (12.7, 23.8) | 135.7 (74.2, 218.9) |
|  | Malawi, 2016 | 748 | B | -0.2 (-7.0, 7.0) | 18.6 (3.5, 35.8) | 3.8 (-2.5, 10.6) | 7.1 (3.0, 11.3) | 18.3 (0.9, 38.7) |
|  |  |  | M | 2.9 (-2.7, 8.9) | 17.2 (2.4, 34.1) | 3.5 (-2.9, 10.3) | 11.2 (7.1, 15.4) | 31.2 (10.4, 56.0) |
|  | Mongolia, 2006 | 239 | B | -3.0 (-15.2, 11.1) | -- | -1.6 (-5.4, 2.4) | -- | 101.1 (30.8, 209.2) |
|  |  |  | M | -2.9 (-14.4, 10.1) | -- | -1.9, -5.6, 2.0) | -- | 70.5 (13.2, 156.6) |
|  | Nicaragua, 2005 | 946 | B | -9.0 (-15.9, -1.6) | -- | 0.7 (-1.9, 3.3) | -- | 113.3 (73.6, 162.2) |
|  |  |  | M | -8.9 (-15.9, -1.2) | -- | 0.4 (-2.1, 3.0) | -- | 116.6 (76.6, 165.7) |
|  | Zambia, 2009 | 330 | B | -8.6 (-18.7, 2.7) | -10.2 (-32.4, 19.3) | 1.4 (-3.1, 6.2) | 8.8 (5.7, 11.9) | 169.4 (101.7, 259.9) |
|  |  |  | M | -8.6 (-18.7, 2.7) | -10.2 (-32.4, 19.3) | 1.4 (-3.1, 6.2) | 8.8 (5.7, 11.9) | 169.4 (101.7, 259.9) |
| Low-middle | Cameroon, 2009 | 556 | B | 12.9 (4.6, 21.9) | 9.3 (-5.1, 25.9) | -0.4 (-2.3, 1.6) | 13.7 (8.8, 18.9) | 172.6 (100.9, 269.9) |
|  |  |  | M | 6.7 (-1.4, 15.4) | 10.7 (-4.4, 28.2) | -0.2 (-2.2, 1.8) | 16.9 (11.8, 22.1) | 203.1 (132.9, 294.5) |
|  | Georgia, 2009 | 2064 | B | 0.3 (-2.5, 3.2) | 2.1 (-7.9, 13.2) | -- | -0.7 (-2.7, 1.3) | -- |
|  |  |  | M | 0.7 (-2.1, 3.5) | 3.1 (-7.0, 14.4) | -- | -0.8 (-2.7, 1.2) | -- |
|  | Nigeria, 2012 | 303 | B | -2.6 (-10.6, 6.1) | 7.9 (-5.5, 23.1) | 0.2 (-2.6, 3.1) | 27.0 (18.1, 36.6) | 262.6 (163.1, 399.8) |
|  |  |  | M | 0.4 (-6.5, 7.8) | 7.9 (-5.5, 23.1) | 0.2 (-2.6, 3.1) | 26.3 (18.2, 35.0) | 246.6 (153.5, 373.9) |
|  | Pakistan, 2011 | 5824 | B | -3.0 (-5.5, -0.5) | -- | 0.6 (-0.4, 1.6) | -- | 22.5 (12.4, 33.6) |
|  |  |  | M | -3.5 (-6.2, -0.8) | -- | 0.7 (-0.4, 1.9) | -- | 23.2 (12.2, 35.4) |
|  | Philippines, 2011 | 1656 | B | -6.2 (-11.9, -0.02) | -11.0 (-20.1, -0.9) | -2.1 (-3.8, -0.3) | 14.0 (10.0, 18.2) | 142.1 (97.6, 196.6) |
|  |  |  | M | -9.9 (-15.6, -3.8) | -9.3 (-18.7, 1.3) | -1.3 (-3.2, 0.5) | 15.7 (11.6, 20.0) | 163.6 (115.8, 222.0) |
|  | Vietnam, 2010 | 344 | B | -11.8 (-17.9, -5.3) | -7.5 (-19.8, 6.6) | -- | 12.7 (5.7, 20.2) | -- |
|  |  |  | M | -11.8 (-17.9, -5.3) | -7.5 (-19.8, 6.6) | -- | 12.7 (5.7, 20.2) | -- |
| Upper-middle | Azerbaijan, 2013 | 987 | B | -2.1 (-6.7, 2.7) | -9.3 (-19.8, 2.5) | -1.9 (-3.9, 0.2) | 15.7 (12.7, 18.7) | 178.7 (137.2, 227.4) |
|  |  |  | M | -0.4 (-5.0, 4.4) | -8.4 (-18.6, 3.2) | -1.5 (-3.6, 0.5) | 14.7 (11.9, 17.6) | 163.7 (125.5, 208.3) |
|  | Colombia, 2010 | 3780 | B | -4.8 (-7.6, -1.9) | -4.2 (-11.7, 4.0) | -- | 6.1 (4.3, 8.0) | -- |
|  |  |  | M | 1.2 (1.0, 1.4) | -5.2 (-12.7, 2.8) | -- | 6.9 (5.1, 8.8) | -- |
|  | Mexico, 2006 | 1562 | B | -3.2 (-8.6, 2.5) | 1.5 (-8.8, 12.9) | -- | 15.8 (10.9, 21.0) | -- |
|  |  |  | M | -3.3 (-8.5, 2.2) | 1.2 (-8.9, 12.4) | -- | 15.6 (10.8, 20.6) | -- |
|  | Mexico, 2012 | 2454 | B | -5.8 (-9.2, -2.2) | 8.0 (-2.7, 19.9) | -- | 10.4 (7.8, 13.0) | -- |
|  |  |  | M | -5.4 (-8.8, -1.9) | 8.1 (-2.6, 20.0) | -- | 10.3 (8.0, 12.8) | -- |
| High | USA, 2006 | 1081 | B | -5.9 (-10.0, -1.6) | 20.3 (8.1, 34.0) | -- | 13.1 (10.1, 16.3) | -- |
|  |  |  | M | -5.4 (-9.5, -1.2) | 21.6 (9.6, 34.9) | -- | 13.5 (10.4, 16.7) | -- |

^1^Ferritin, CRP and AGP variables were *natural-log* transformed for analysis due to non-normal distributions. Regression estimates were exponentiated, and results are presented as the percent change (95% confidence interval) in the dependent variable for every 1-unit change in the independent variable. Note that for the values presented for ‘Ferritin regressed on *ln*CRP’ and ‘Ferritin regressed on *ln*AGP’, the percent changes in ferritin concentration are for every 1-unit change in *natural-log* transformed CRP or AGP. All estimates account for cluster survey design (cluster, strata) with survey weights applied, except in the Mongolia survey which followed a simple random sampling design. Ferritin was measured in either serum or plasma, as reported by the survey. Covariates available for adjustment were: age, education level (maternal or household head), household socioeconomic status, access to an improved water source, access to an improved toilet, and urban/rural residence. Covariates were included in the multivariable regression model if they were associated with the outcome variable at p<0.1 in the bivariate model. Inclusion criteria were: BAZ or WHZ ≥ -2 SD and a negative malaria test result. Country income classification was defined according to the World Bank definition for the year in which the survey was conducted (4). ‘--‘ indicates the variable was unavailable in that survey. AGP, α-1-acid glycoprotein; B, bivariate model; BAZ, BMI-for-age z-score; BRINDA, Biomarkers Reflecting Inflammation and Nutritional Determinants of Anemia; CRP, C-reactive protein; M, multivariable model; WHZ, weight-for-height z-score.

**Supplemental Table 9.** Unadjusted and adjusted associations between ferritin, CRP or AGP, and BAZ stratified by age among preschool-age children (6-59 months) with normal weight to overweight/obesity by survey: BRINDA project^1^

|  |  |  | **Stratified analysis, unadjusted** | | **Stratified analysis, adjusted** | |
| --- | --- | --- | --- | --- | --- | --- |
|  |  |  | β (95% confidence interval) | | β (95% confidence interval) | |
| **Survey, year** | **n** | **Model** | **Ages 6-23 months** | **Ages 24-59 months** | **Ages 6-23 months** | **Ages 24-59 months** |
| Afghanistan, 2013 | 595 | No effect modification | -- | -- | -- | -- |
| Bangladesh, 2010 | 1179 | Survey only included children 6-24 mo. No stratification possible. | -- | -- | -- | -- |
| Bangladesh, 2012 | 368 | No effect modification | -- | -- | -- | -- |
| Burkina Faso, 2010 | 63 | All children older than 24 months No stratification possible. | -- | -- | -- | -- |
| Cambodia, 2014 | 599 | *ln*CRP = BAZ | -0.23 (-0.5, 0.05) | 0.22 (0.03, 0.41) | -0.23 (-0.5, 0.04) | 0.22 (0.03, 0.41) |
| Côte d’Ivoire, 2007 | 435 | *ln*CRP = BAZ | -0.15 (-0.43, 0.12) | 0.27 (0.1, 0.44) | -0.15 (-0.43, 0.12) | 0.26 (0.1, 0.41) |
|  |  | *ln*AGP = BAZ | -0.04 (-0.08, -0.01) | 0.03 (-0.02, 0.08) | -0.05 (-0.09, -0.01) | 0.02 (-0.02, 0.07) |
| Kenya, 2007 | 665 | *ln*Ferritin = BAZ | -0.14 (-0.25, -0.03) | 0.05 (-0.08, 0.17) | -0.12 (-0.23, 0.01) | 0.04 (-0.1, 0.18) |
| Kenya, 2010 | 551 | No effect modification | -- | -- | -- | -- |
| Laos, 2006 | 443 | *ln*Ferritin = BAZ | 0.23 (0.02, 0.44) | -0.17 (-0.30, -0.40) | 0.19 (-0.01, 0.40) | -0.14 (-0.25, -0.02) |
| Liberia, 2011 | 956 | *ln*Ferritin = *log*AGP | 1.07 (0.75, 1.39) | 0.48 (0.05, 0.92) | No covariates to test. | |
| Malawi, 2016 | 748 | No effect modification | -- | -- | -- | -- |
| Mongolia, 2006 | 239 | No effect modification | -- | -- | -- | -- |
| Nicaragua, 2005 | 946 | *ln*AGP = BAZ | -0.04 (-0.09, 0.01) | 0.03 (0.005, 0.05) | -0.04 (-0.09, 0.01) | 0.03 (0.002, 0.05) |
|  |  | *ln*Ferritin = *ln*AGP | 1.02 (0.59, 1.45) | 0.63 (0.44, 0.82) | No covariates to test. | |
| Zambia, 2009 | 330 | No effect modification | -- | -- | -- | -- |
| Cameroon, 2009 | 556 | *ln*Ferritin = *ln*AGP | 1.49 (0.96, 2.02) | 0.89 (0.5, 1.27) | 1.31 (0.8, 1.81) | 0.87 (0.48, 1.27) |
| Georgia, 2009 | 2065 | No effect modification | -- | -- | -- | -- |
| Nigeria, 2012 | 303 | *ln*AGP = BAZ | -0.01 (-0.09, 0.07) | 0.004 (-0.02, 0.03) | No covariates to test | |
|  |  | *ln*Ferritin= *ln*CRP | 0.24 (0.11, 0.37) | 0.24 (0.19, 0.29) | No covariates to test | |
| Pakistan, 2011 | 5824 | No effect modification | -- | -- | -- | -- |
| Philippines, 2011 | 1656 | All children older than 24 months No stratification possible. | -- | -- | -- | -- |
| Vietnam, 2010 | 344 | *ln*Ferritin=*ln*CRP | 0.24 (0.11, 0.38) | 0.11 (0.04, 0.17) | No covariates to test | |
| Azerbaijan, 2013 | 987 | *ln*Ferritin=BAZ | -0.09 (-0.18, -0.0006) | 0.01 (-0.04, 0.07) | -0.09 (-0.18, 0.001) | 0.01 (-0.04, 0.07) |
|  |  | *ln*Ferritin=*ln*CRP | 0.08 (0.02, 0.14) | 0.16 (0.14, 0.19) | 0.08 (0.02, 0.14) | 0.16 (0.13, 0.18) |
| Colombia, 2010 | 3780 | *ln*Ferritin = BAZ | -0.12 (-0.18, -0.05) | -0.02 (-0.05, 0.02) | No covariates to test | |
| Mexico, 2006 | 1562 | *ln*Ferritin=*ln*CRP | 0.04 (-0.07, 0.15) | 0.16 (0.11, 0.20) | No covariates to test | |
| Mexico, 2012 | 2454 | No effect modification | -- | -- | -- | -- |
| United States, 2006 | 1081 | *ln*Ferritin=*ln*CRP | 0.21 (0.15, 0.26) | 0.1 (0.06, 0.13) | 0.21 (0.15, 0.27) | 0.1 (0.06, 0.13) |

^1^Results presented as the unexponentiated β (95% confidence interval). Effect modification was evaluated through stratified analyses in each of the bivariate models listed in the table by testing for a significant interaction between the predictor (i.e., BAZ, *ln*CRP, or *ln*AGP) and the effect modifier variable of age. Unadjusted stratified analyses were completed for all models where p<0.1 for the interaction. Adjusted stratified analyses were then completed for any marginally significant covariates (p<0.1) found in bivariate analyses. All estimates account for the complex survey design (cluster and strata) with survey weights applied, except in the survey from Mongolia which followed a simple random sampling design. Covariates available for adjustment were: education level (maternal or household head), household socioeconomic status, access to an improved water source, access to an improved toilet, and urban/rural residence. Inclusion criteria were: BAZ or WHZ ≥-2 SD and a negative malaria test result. AGP, α-1-acid glycoprotein; BAZ, BMI-for-age z-score; BRINDA, Biomarkers Reflecting Inflammation and Nutritional Determinants of Anemia; CRP, C-reactive protein; WHZ, weight-for-height z-score.

**Supplemental Table 10.** Unadjusted and adjusted associations between ferritin, CRP or AGP, and BAZ stratified by sex among preschool age children (6-59 months) with normal weight to overweight/obesity by survey: BRINDA project^1^

|  |  |  | **Stratified analysis, unadjusted** | | **Stratified analysis, adjusted** | |
| --- | --- | --- | --- | --- | --- | --- |
|  |  |  | β (95% confidence interval) | | β (95% confidence interval) | |
| **Survey, year** | **n** | **Model** | **Male** | **Female** | **Male** | **Female** |
| Afghanistan, 2013 | 595 | *ln*AGP = BAZ | -0.07 (-0.10, -0.03) | -0.01 (0.61, -0.05) | -0.07 (-0.10, -0.03) | -0.01 (0.61, -0.05) |
| Bangladesh, 2010 | 1179 | No effect modification | -- | -- | -- | -- |
| Bangladesh, 2012 | 368 | *ln*Ferritin=*ln*CRP | 0.18 (0.08, 0.28) | 0.3 (0.22, 0.37) | 0.2 (0.12, 0.29) | 0.25 (0.17, 0.33) |
| Burkina Faso, 2010 | 63 | No effect modification | -- | -- | -- | -- |
| Cambodia, 2014 | 599 | No effect modification | -- | -- | -- | -- |
| Côte d’Ivoire, 2007 | 435 | No effect modification | -- | -- | -- | -- |
| Kenya, 2007 | 665 | *ln*CRP = BAZ | -0. 1 (-0.26, 0.05) | 0.19 (-0.04, 0.43) | -0.06 (-0.21, 0.08) | 0.2 (-0.04, 0.44) |
| Kenya, 2010 | 551 | *ln*AGP = BAZ | -0.01 (-0.04, 0.03) | 0.04 (-0.002, 0.09) | -0.01 (-0.05, 0.03) | 0.04 (-0.01, 0.08) |
| Laos, 2006 | 443 | No effect modification | -- | -- | -- | -- |
| Liberia, 2011 | 956 | No effect modification | -- | -- | -- | -- |
| Malawi, 2016 | 748 | *ln*CRP = BAZ | 0.05 (-0.15, 0.24) | 0.36 (0.14, 0.57) | 0.05 (-0.14, 0.24) | 0.34 (0.13, 0.56) |
| Mongolia, 2006 | 239 | No effect modification | -- | -- | -- | -- |
| Nicaragua, 2005 | 946 | *ln*AGP = BAZ | -0.02 (-0.06, 0.02) | 0.03 (-0.003, 0.06) | -0.02 (-0.06, 0.02) | 0.03 (-0.004, 0.06) |
|  |  | *ln*Ferritin=*ln*AGP | 0.73 (0.4, 1.06) | 0.79 (0.52, 1.06) | 0.73 (-0.4, 1.07) | 0.82 (0.56, 1.08) |
| Zambia, 2009 | 330 | No effect modification | -- | -- | -- | -- |
| Cameroon, 2009 | 556 | No effect modification | -- | -- | -- | -- |
| Georgia, 2009 | 2065 | *ln*Ferritin=*ln*CRP | -0.02 (-0.04, 0.004) | 0.004 (-0.02, 0.03) | -0.02 (-0.04, 0.002) | 0.004 (-0.02, 0.03) |
| Nigeria, 2012 | 303 | No effect modification | -- | -- | -- |  |
| Pakistan, 2011 | 5824 | *ln*Ferritin=*ln*AGP | 0.19 (0.07, 0.30) | 0.22 (0.09, 0.34) | 0.19 (0.07, 0.32) | 0.22 (0.08, 0.35) |
| Philippines, 2011 | 1656 | No effect modification | -- | -- | -- | -- |
| Vietnam, 2010 | 344 | No effect modification | -- | -- | -- | -- |
| Azerbaijan, 2013 | 987 | No effect modification | -- | -- | -- | -- |
| Colombia, 2010 | 3780 | No effect modification | -- | -- | -- | -- |
| Mexico, 2006 | 1562 | No effect modification | -- | -- | -- | -- |
| Mexico, 2012 | 2454 | No effect modification | -- | -- | -- | -- |
| United States, 2006 | 1081 | No effect modification | -- | -- | -- | -- |

^1^Results presented as the unexponentiated β (95% confidence interval). Effect modification was evaluated through stratified analyses in each of the bivariate models listed in the table by testing for a significant interaction between the predictor (i.e., BAZ, *ln*CRP, or *ln*AGP) and the effect modifier variable of sex. Unadjusted stratified analyses were completed for all models where p<0.1 for the interaction. Adjusted stratified analyses were then completed for any marginally significant covariates (p<0.1) found in bivariate analyses. All estimates account for the complex survey design (cluster and strata) with survey weights applied, except in the survey from Mongolia which followed a simple random sampling design. Covariates available for adjustment were: education level (maternal or household head), household socioeconomic status, access to an improved water source, access to an improved toilet, and urban/rural residence. Inclusion criteria were: BAZ or WHZ ≥-2 SD and a negative malaria test result. AGP, α-1-acid glycoprotein; BAZ, BMI-for-age z-score; BRINDA, Biomarkers Reflecting Inflammation and Nutritional Determinants of Anemia; CRP, C-reactive protein; WHZ, weight-for-height z-score.

**Supplemental Table 11.** Unadjusted relationships between ferritin and BMI or BAZ as mediated by inflammation among women of reproductive age (15-49 years) and preschool-age children (6-59 months) with normal weight to overweight/obesity by survey: BRINDA project^1^

|  |  |  | **WRA mediation analysis, unadjusted^2^** | | | | | |
| --- | --- | --- | --- | --- | --- | --- | --- | --- |
| **Country income classification^3^** | **Country, survey year** | **n** | **Total Effect** | **Direct Effect** | **Indirect Effect** | **% Mediated** | **% Mediated by CRP** | **% Mediated by AGP** |
| Low | Afghanistan, 2013 | 571 | -2.1 (-4.3, 0.1) | -2.7 (-4.7,- 0.8) | 0.6 (-0.02, 1.3) | NM | -- | -- |
|  | Burkina Faso, 2010 | 61 | -2.9 (-15.9, 10.1) | 5.1 (-6.1, 17.2) | -7.5 (-12.5, -3.2) | NM | -- | -- |
|  | Cambodia, 2014 | 609 | 3.8 (2.0, 5.5) | 2.3 (0.4, 4.1) | 1.4 (0.2, 2.6) | 38% | 17% | 21% |
|  | Côte d’Ivoire, 2007 | 706 | 0.2 (-1.4, 1.8) | -1.0 (-2.6, 0.5) | 1.3 (0.8, 1.8) | NM | -- | -- |
|  | Laos, 2006 | 690 | 11.9 (8.6, 15.3) | 9.1 (5.7, 12.7) | 2.6 (1.2, 4.0) | 23%^4^ | 17% | 3% |
|  | Malawi, 2016 | 594 | -0.5 (-2.5, 1.6) | -1.1 (-3.2, 0.1) | 0.6 (0.04, 1.2) | NM | -- | -- |
| Low-middle | Cameroon, 2009 | 594 | -0.01 (-1.3, 1.3) | -0.7 (-1.9, 0.5) | 0.7 (0.4, 1.0) | NM | -- | -- |
|  | Georgia, 2009 | 1605 | 1.2 (0.6, 1.8) | 0.8 (0.2, 1.4) | 0.4 (0.1, 0.6) | 31% | 31% | -- |
|  | India, 2011 | 147 | 7.5 (0.07, 15.4) | 3.7 (-2.1, 9.4) | 3.6 (1.1, 6.2) | NM^5^ | -- | -- |
|  | Nigeria, 2012 | 506 | -1.5 (-3.2, 0.3) | -1.5 (-3.2, 0.2) | 0.003 (-0.04, 0.5) | NM | -- | -- |
|  | Pakistan, 2011 | 5004 | 1.0 (0.4, 1.7) | 0.8 (0.2, 1.5) | 0.2 (0.1, 0.3) | 21% | 9% | 11% |
|  | Vietnam, 2010 | 1178 | 4.1 (1.7, 6.7) | 1.1 (-1.4, 3.7) | 3.0 (2.1, 3.9) | 73% | 73% | -- |
| Upper-middle | Azerbaijan, 2013 | 2528 | 3.5 (2.7, 4.3) | 1.4 (0.1, 2.3) | 2.1 (1.6, 2.6) | 60% | 45% | 15% |
|  | Colombia, 2010 | 8300 | 2.0 (1.4, 2.6) | 1.6 (1.0, 2.2) | 0.4 (0.2, 0.5) | 19% | 19% | -- |
|  | Mexico, 2006 | 2910 | 1.8 (0.8, 2.8) | 0.8 (-0.3, 1.8) | 1.0 (0.4, 1.6) | 58% | 58% | -- |
|  | Mexico, 2012 | 3540 | 2.7 (1.3, 4.2) | 0.8 (-1.0, 2.5) | 2.0 (1.3, 2.6) | 71% | 71% | -- |
| High | United Kingdom, 2014 | 862 | 1.9 (0.5, 3.3) | 1.3 (-1.0, 2.9) | 0.6 (-0.04, 1.2) | NM | -- | -- |
|  | United States, 2006 | 3024 | 1.3 (0.6, 2.0) | -0.03 (-1.0, 0.08) | 1.3 (1.0, 1.7) | 100% | 100% | -- |
|  |  |  | **PSC mediation analysis, unadjusted^2^** | | | | | |
| Low | Afghanistan, 2013 | 595 | -0.9 (-2.8, 1.1) | -0.6 (-9.9, 8.7) | -1.4 (-10.5, 7.6) | NM | -- | -- |
|  | Bangladesh, 2010 | 1179 | -6.5 (-11.0, -2.3) | -5.7 (-10.1, -1.6) | -0.8 (-2.2, 0.5) | NM | -- | -- |
|  | Bangladesh, 2012 | 368 | -4.8 (-15.3, 5.5) | -2.7 (-12.6, 7.1) | -2.2 (-6.1, 1.7) | NM | -- | -- |
|  | Burkina Faso, 2010 | 63 | -10.5 (-41.9, 19.8) | -10.6 (-37.8, 15.4) | 0.1 (-7.5, 7.7) | NM | -- | -- |
|  | Cambodia, 2014 | 599 | -6.2 (-16.6, 3.8) | -7.0 (-16.6, 2.0) | 0.9 (-3.3, 5.0) | NM | -- | -- |
|  | Côte d’Ivoire, 2007 | 435 | 1.4 (-7.2, 10.0) | -0.6 (-8.6, 7.4) | 2.0 (-2.2, 6.2) | NM | -- | -- |
|  | Kenya, 2007 | 665 | -7.3 (-16.8, 1.6) | -7.2 (-16.0, 1.1) | -0.1 (-2.7, 2.5) | NM | -- | -- |
|  | Kenya, 2010 | 551 | -5.0 (-13.4, 3.2) | -7.3 (-15.0, -0.2) | 2.5 (-1.8, 6.7) | NM | -- | -- |
|  | Laos, 2006 | 443 | -5.8 (-18.2, 6.2) | -4.5 (-17.2, 7.9) | -1.3 (-5.0, 2.3) | NM | -- | -- |
|  | Liberia, 2011 | 956 | -1.6 (-6.9, 3.7) | -1.7 (-7.2, 3.8) | 0.1 (-1.9, 2.1) | NM | -- | -- |
|  | Malawi, 2016 | 748 | -3.0 (-16.4, 10.4) | -1.5 (-8.5, 5.4) | 1.3 (-1.5, 4.0) | NM | -- | -- |
|  | Mongolia, 2006 | 239 | -0.2 (-7.2, 6.7) | -1.9 (-15.0, 11.2) | -1.1 (-3.9, 1.7) | NM | -- | -- |
|  | Nicaragua, 2005 | 946 | -9.0 (-17.2, -1.7) | -9.5 (-16.8, -3.1) | 0.5 (-1.4, 2.4) | NM | -- | -- |
|  | Zambia, 2009 | 330 | -8.6 (-20.1, 2.0) | -9.2 (-20.0, 0.8) | 0.6 (-4.1, 5.4) | NM | -- | -- |
| Low-middle | Cameroon, 2009 | 556 | 12.9 (4.6, 19.7) | 12.5 (4.9, 18.7) | 0.3 (-1.8, 2.5) | NM | -- | -- |
|  | Georgia, 2009 | 2064 | 0.3 (-2.5, 3.1) | 0.3 (-2.5, 3.1) | -0.02 (-0.1, 0.1) | NM | -- | -- |
|  | Nigeria, 2012 | 303 | -2.6 (-10.8, 5.5) | -3.8 (-13.2, 5.5) | 1.2 (-2.2, 4.7) | NM | -- | -- |
|  | Pakistan, 2011 | 5824 | -3.0 (-5.6, -0.5) | -3.1 (-5.8, -0.6) | 0.1 (-0.1, 0.3) | NM | -- | -- |
|  | Philippines, 2011 | 1656 | -6.2 (-12.6, -0.1) | -4.3 (-10.7, 1.9) | -2.0 (-3.7, -0.2) | 31% | 8% | 23% |
|  | Vietnam, 2010 | 344 | -11.8 (-19.4, -5.8) | -11.1 (-19.1, -4.3) | -0.9 (-2.5, 0.7) | NM | -- | -- |
| Upper-middle | Azerbaijan, 2013 | 987 | -2.1 (-6.9, 2.7) | -0.1( -4.6, 4.3) | -2.0 (-4.2, 0.2) | NM | -- | -- |
|  | Colombia, 2010 | 3780 | -4.8 (-7.9, -1.9) | -4.7 (-7.8, -1.8) | -0.2 (-0.6, 0.3) | NM | -- | -- |
|  | Mexico, 2006 | 1562 | -3.2 (-9.0, 2.5) | -3.4 (-9.3, 2.4) | 0.2 (-1.3, 1.8) | NM | -- | -- |
|  | Mexico, 2012 | 2454 | -5.8 (-9.6, -2.3) | -6.5 (-10.3, -3.2) | 0.8 (-0.3, 1.8) | NM | -- | -- |
| High | USA, 2006 | 1081 | -5.8 (-9.6, -2.3) | -6.5 (-10.3, -3.2) | 0.8 (-0.3, 1.8) | NM | -- | -- |

^1^Mediation effects were exponentiated and results are presented as percent change (95% confidence interval) in ferritin for every 1-unit change in BMI (WRA) or BAZ (PSC). Ferritin concentration measured in serum or plasma, as reported in the survey. All estimates account for cluster survey design (cluster, strata) with survey weights applied, except in the survey from Mongolia which used simple random sampling. Inclusion criteria were: BMI ≥18.5 kg/m2 (WRA) or BAZ or WHZ ≥-2 SD (PSC), not pregnant (WRA only), and a negative malaria result. AGP, alpha-1-acid glycoprotein; BAZ, BMI-for-age z-score; BMI, body mass index; BRINDA, Biomarkers Reflecting Inflammation and Nutritional Determinants of Anemia; CRP, C-reactive protein; NM, no mediation; PSC, pre-school age children; WHZ, weight-for-height z-score; WRA, women of reproductive age.

^2^Model for mediation analysis: *ln*Ferritin = β_0_ + β_1_(BMI or BAZ) + *M*_1_(*ln*CRP) [+*M*_2_(*ln*AGP)] where all values were continuous and AGP was included as a mediator only in analyses for which it was available in the dataset. Interpretation is as follows: Total Effect = the effect of BMI (or BAZ) on ferritin; Direct Effect = the effect of BMI (or BAZ) on ferritin controlling for inflammation; Indirect Effect = the effect of BMI (or BAZ) on ferritin as mediated by the effect of CRP or AGP. Mediation was considered present when both the total and indirect effects were significant (5).

^3^Country income classification defined according to the World Bank definition for the year in which the survey was conducted (4).

^4^In the survey from Laos (WRA), 23% of the relationship between BMI and ferritin was mediated by inflammation, with 17% of the mediated effect through CRP, 3% of the mediated effect through AGP, and 3% of the mediated effect unexplained.

^5^For the survey from India (WRA), the confidence interval for the total effect appears significant, however as the p-value was 0.063 mediation was not considered present.

**Supplemental Table 12.** Sensitivity analysis: mediation analysis (unadjusted) assessing the relationship between ferritin, BMI or BAZ and inflammation including and excluding observations that tested positive for malaria for WRA and PSC with normal weight to overweight/obesity: BRINDA project^1^

|  | **Including positive malaria observations (sensitivity analysis)** | | **Excluding positive malaria observations (original analysis)** | |
| --- | --- | --- | --- | --- |
| Survey, year | n | β (95% CI) | n | β (95% CI) |
| *WRA* |  | | | |
| Cameroon, 2009 | 691 | 0.005 (0.002, 0.008) | 594 | 0.01 (0.004, 0.01) |
| Côte d’Ivoire, 2007 | 742 | 0.01 (0.01, 0.02) | 706 | 0.01 (0.01, 0.02) |
| Malawi, 2016 | 693 | 0.007 (0.003, 0.01) | 594 | 0.01 (0.0004, 0.01) |
| Nigeria, 2012 | 555 | -0.0002 (-0.005, 0.004) | 506 | 0.00003 (-0.005, 0.005) |
| *PSC* |  | | | |
| Cameroon, 2009 | 740 | -0.005 (-0.04, 0.03) | 556 | 0.003 (-0.02, 0.02) |
| Côte d’Ivoire, 2007 | 606 | 0.01 (-0.03, 0.06) | 435 | 0.02 (-0.02, 0.06) |
| Kenya, 2007 | 825 | 0.01 (-0.02, 0.04) | 665 | -0.001 (-0.03, 0.03) |
| Kenya, 2010 | 813 | 0.01 (-0.03, 0.06) | 551 | 0.03 (-0.02, 0.07) |
| Liberia, 2011 | 1268 | 0.01 (-0.02, 0.05) | 956 | 0.001 (-0.02, 0.02) |
| Malawi, 2016 | 1027 | 0.04 (0.004, 0.07) | 748 | 0.01 (-0.02, 0.04) |
| Nigeria, 2012 | 452 | 0.02 (-0.02, 0.06) | 303 | 0.01 (-0.02, 0.05) |
| Zambia, 2009 | 406 | -0.006 (-0.06, 0.05) | 330 | 0.01 (-0.04, 0.05) |

^1^Estimates of the mediated effect are presented as the unexponentiated β coefficient of *ln*Ferritin (95% confidence intervals). β represents the indirect effect, that is the effect of BMI or BAZ on ferritin concentration as mediated by the effect of CRP or AGP. Malaria status was evaluated by survey-specific diagnostic tests that have been previously described (3). While Burkina Faso (2010) measured malaria status, all observations for both WRA and PSC were excluded as part of the criteria to exclude observations with underweight/wasting, thus the survey is not included in this sensitivity analysis. BAZ, BMI-for-age z-score; BMI, body mass index; BRINDA, Biomarkers Reflecting Inflammation and Nutritional Determinant of Anemia; PSC, preschool-age children (6-59 months); WRA, women of reproductive age (15-49 years).

**References**

1. Williams AM, Guo J, Addo OY, Ismaily S, Namaste SML, Oaks BM, Rohner F, Suchdev PS, Young MF, Flores-Ayala R, et al. Intraindividual double burden of overweight or obesity and micronutrient deficiencies or anemia among women of reproductive age in 17 population-based surveys. Am J Clin Nutr. 2020 Aug 1;112(Supplement_1):468S-477S.

2. Engle-Stone R, Guo J, Ismaily S, Addo OY, Ahmed T, Oaks B, Suchdev PS, Flores-Ayala R, Williams AM. Intraindividual double burden of overweight and micronutrient deficiencies or anemia among preschool children. Am J Clin Nutr. 2020 Aug 1;112(Supplement_1):478S-487S.

3. Namaste SM, Aaron GJ, Varadhan R, Peerson JM, Suchdev PS. Methodologic approach for the Biomarkers Reflecting Inflammation and Nutritional Determinants of Anemia (BRINDA) project. Am J Clin Nutr. 2017 Jul 1;106(suppl_1):333S-347S.

4. The World Bank. World Development Indicators - The World by Income and Region [Internet]. [cited 2021 Apr 29]. Available from: https://datatopics.worldbank.org/world-development-indicators/the-world-by-income-and-region.html

5. Baron RM, Kenny DA. The moderator-mediator variable distinction in social psychological research: conceptual, strategic, and statistical considerations. J Pers Soc Psychol. 1986;51(6):1173–82.
